# Bifunctional Electrocatalysts with High-Entropy Alloys: Bridging Hydrogen Evolution and Oxygen Reduction

**DOI:** 10.1021/acs.chemmater.6c00035

**Published:** 2026-04-01

**Authors:** Jialu Li, Jianzhuo Wu, Abdulrahman Allangawi, Yu Li, Honglin Li, Yongfeng Guo, Huabin Zhang, Wan-Lu Li

**Affiliations:** † Program of Materials Science and Engineering, 8784 University of California San Diego, La Jolla, California 92093, United States; ‡ Aiiso Yufeng Li Family Department of Chemical and Nano Engineering, 8784 University of California San Diego, La Jolla, California 92093, United States; § Center for Renewable Energy and Storage Technologies (CREST), Physical Science and Engineering Division, King Abdullah University of Science and Technology (KAUST), Thuwal 23955-6900, Saudi Arabia

## Abstract

High-entropy alloys (HEAs) have emerged as a promising class of bifunctional electrocatalysts capable of simultaneously driving the hydrogen evolution reaction (HER) and the oxygen reduction reaction (ORR) with high activity and durability. Their near-equiatomic multicomponent compositions give rise to unique physicochemical characteristics, including lattice distortion, sluggish diffusion, high-entropy stabilization, and pronounced electronic heterogeneity, that collectively generate diverse and synergistic active sites inaccessible in conventional alloys. This review summarizes recent progress in HEA-based bifunctional electrocatalysis, with a focus on the fundamental mechanisms governing HER and ORR activity, stability, and selectivity. We discuss advances in synthesis strategies, ranging from confined growth and step-alloying to scalable continuous-flow methods, that enable precise control over composition, size, and surface structure. Complementary computational and data-driven approaches, including density functional theory, machine-learning-assisted screening, and descriptor development, are highlighted as essential tools for navigating the vast HEA design space and establishing structure–property relationships. Particular attention is paid to adsorption-energy distributions, multisite cooperativity, and environmental effects under realistic electrochemical conditions. Finally, we outline current challenges and future opportunities for integrating mechanistic understanding with AI-guided, closed-loop design frameworks to accelerate the discovery of next-generation HEA bifunctional electrocatalysts for sustainable energy conversion.

## Graphical abstract

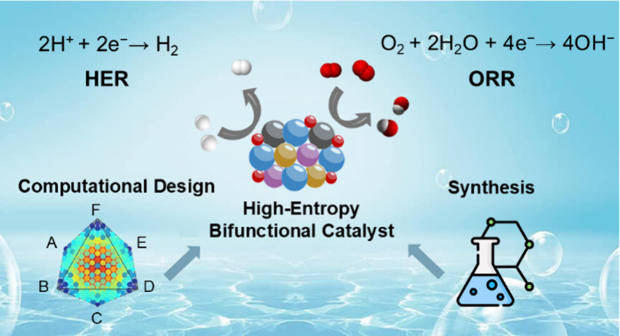

## 1 Introduction

The growing global demand for environmentally sustainable and carbon-neutral energy conversion technologies has driven extensive research into efficient electrocatalysts for key electrochemical reactions, most notably the hydrogen evolution reaction (HER) and the oxygen reduction reaction (ORR).^1–6^ HER underpins hydrogen production in water-splitting systems, while ORR largely governs the performance of fuel cells and metal–air batteries through its multielectron reaction pathway.^7–9^ Despite substantial advances, the reliance on noble-metal catalysts remains a major bottleneck for scalable implementation. State-of-the-art catalysts based on Pt- and Ru-group metals exhibit high activity but suffer from high cost, limited elemental abundance, and durability concerns under operating conditions.^10–14^ Consequently, the development of stable, cost-effective bifunctional catalysts capable of efficiently catalyzing both HER and ORR remains a central challenge in electrochemical energy research.

High-entropy alloys (HEAs) have attracted increasing attention as catalytic materials owing to their distinctive physicochemical characteristics arising from compositional complexity.^15–17^ Unlike conventional alloys dominated by one or two elements, HEAs are composed of multiple principal elements in near-equiatomic ratios, resulting in intrinsically disordered atomic configurations.^18,19^ This compositional disorder gives rise to several characteristic effects such as lattice distortion, sluggish diffusion, high-entropy stabilization, and the cocktail effect, which collectively enhance structural stability, electronic heterogeneity, and a broad distribution of local atomic environments.^20–22^ Also, such diversity enables the modulation of adsorption energies for key reaction intermediates (*H, *O, *OH, *OOH) across distinct surface sites, providing a materials-level basis for accommodating the differing adsorption requirements of HER and ORR within a single catalyst.^5,11,23^ At the nanoscale, these intrinsic features can be further leveraged through controlled synthesis and compositional engineering.^19^ Recent progress in confined growth, step-alloying, and continuous-flow synthesis has enabled the preparation of HEA nanoparticles with well-defined size, composition, and elemental dispersion.^24–26^ In such systems, multicomponent surfaces can host spatially differentiated active motifs, such as oxophilic sites that facilitate water activation and metallic sites that favor hydrogen or oxygen intermediate conversion. In parallel, compositional tuning induces systematic electronic modulation, including shifts in the d-band center, which governs adsorption–desorption energetics.^2,27^ Together, nanoscale structure control and electronic heterogeneity position HEAs as a versatile platform for exploring bifunctional electrocatalysis beyond the limitations of conventional noble-metal catalysts.^28^ Despite these advantages, the rational design of HEA-based bifunctional catalysts remains challenging. The vast compositional space and intrinsic atomic-scale disorder complicate the identification of clear structure–property relationships, while dynamic surface reconstruction and cooperative effects under electrochemical operating conditions are not yet well understood. Addressing these challenges requires an integrated approach that combines controlled synthesis, theoretical modeling, and data-driven analysis to resolve mechanistic pathways and guide the targeted discovery of high-performance HEAs for HER and ORR.^29–32^

This review summarizes recent advances in the design, synthesis, and computational understanding of high-entropy alloy catalysts for bifunctional electrocatalysis. We first outline the key physicochemical effects and synergistic mechanisms that govern catalytic behavior in HEAs, followed by an assessment of their bifunctional performance toward HER and ORR. We then discuss emerging synthesis strategies and computational methodologies that enable precise structural control and predictive performance evaluation across the vast HEA compositional space. Finally, we highlight current challenges and future opportunities for data-driven and mechanism-guided HEA design, offering perspectives on the development of next-generation bifunctional electrocatalysts for sustainable energy conversion.

## 2 Physicochemical Origins of Catalytic Activity in High-Entropy Alloys

### 2.1 Core Effects of HEAs

HEAs are composed of multiple principal elements in near-equiatomic proportions, resulting in physicochemical characteristics that differ fundamentally from those of conventional single- or binary-metal catalysts. Rather than being governed by a dominant elemental component, HEAs derive their structural stability and catalytic behavior from collective effects associated with compositional complexity. These behaviors are commonly rationalized in terms of four interrelated core effects (Figure 1): lattice distortion, sluggish diffusion, high-entropy stabilization, and the cocktail effect.^18–20,22,33^

**Figure 1:**
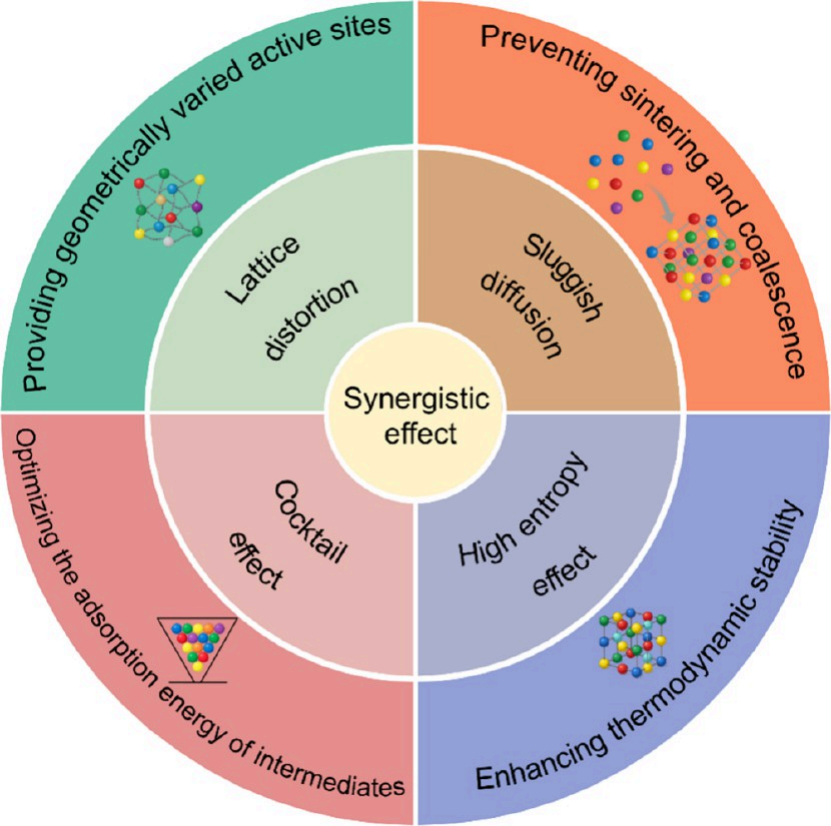
Schematic illustration of the four core effects and the synergistic effect that define the advantages of HEAs in electrocatalysis.

Lattice distortion arises from the mismatch in atomic radii and bonding preferences among constituent elements, leading to local strain fields and deviations from ideal lattice symmetry.^22^ At the atomic scale, such distortions generate a wide spectrum of coordination environments and bond lengths at both bulk and surface sites.^20^ This structural heterogeneity alters orbital overlap and local electronic density, directly influencing the adsorption energetics of reaction intermediates. Consequently, it produces a distribution of active sites with varying affinities toward *H, *O, *OH, and *OOH species.^5^ As a result, HEA surfaces can support multiple reaction steps across different sites, a feature that is particularly advantageous for complex, multistep electrocatalytic processes.^33^

Sluggish diffusion is a kinetic consequence of severe chemical disorder, where the presence of multiple elements with different migration barriers suppresses long-range atomic mobility.^19^ This effect inhibits grain growth, surface coalescence, and elemental segregation during synthesis and electrochemical operation.^34^ From a catalytic standpoint, sluggish diffusion contributes to enhanced structural robustness by stabilizing nanoscale features and preserving active surface motifs under prolonged cycling, elevated temperatures, or highly oxidative or reductive environments.

High-entropy stabilization originates from the large configurational entropy associated with multicomponent solid solutions, which thermodynamically favors homogeneous disordered phases over ordered intermetallic compounds.^18^ This stabilization effect plays a crucial role in suppressing phase separation and maintaining uniform elemental distributions, particularly under nonequilibrium conditions encountered during electrocatalysis.^20^ By stabilizing single-phase structures across broad compositional ranges, high-entropy stabilization provides a reliable platform for systematic composition tuning while preserving long-term catalytic durability.^5^

The cocktail effect captures the nonlinear and often nonadditive interactions among different elemental components in HEAs.^20,22^ Through combined ligand effects, charge redistribution, and orbital hybridization, the presence of multiple elements enables continuous tuning of electronic structure parameters, such as the d-band center and density of states near the Fermi level.^5^ These electronic variations reshape the adsorption-energy landscape across the surface, allowing catalytic activity and selectivity to be adjusted beyond what is achievable in single-component systems. Importantly, the cocktail effect does not imply a simple averaging of elemental properties but rather the emergence of new catalytic behavior arising from multicomponent interactions.^20^

### 2.2 Synergistic Interactions in Multicomponent HEA Catalysts

Beyond the intrinsic physicochemical effects discussed above, synergistic interactions among multiple elements play a central role in governing the bifunctional electrocatalytic behavior of HEAs. These interactions arise from atomic-scale coupling between chemically distinct elements and manifest through several interconnected mechanisms, including electronic modulation, strain-induced geometric effects, and cooperative adsorption.^23^

Electronic synergy originates from interactions between elements with different electronegativities and d-band filling characteristics, leading to charge redistribution within the alloy.^35^ This redistribution modifies the local d-band center of surface atoms and directly influences the adsorption energies of key intermediates such as *H, *OH, and *OOH, thereby affecting both HER and ORR activity.^36^ For example, alloying Pt with Ni induces a downward shift of the Pt d-band center, weakening hydrogen binding and improving reaction kinetics relative to monometallic Pt.^37^

Geometric and strain-related effects arise from differences in atomic radii among constituent elements, which introduce local lattice strain and distort coordination environments at the surface. These distortions generate a diversity of adsorption geometries, such as locally under- or overcoordinated sites, that can stabilize different intermediates along multistep reaction pathways.^38,39^ Such geometric heterogeneity is particularly important for multielectron processes like ORR, where sequential adsorption and transformation of oxygenated species are required.^11^

In addition, HEAs enable cooperative adsorption through the coexistence of chemically distinct surface sites. Different reaction steps can occur preferentially on different elemental motifs.^5^ For example, water activation on oxophilic sites and hydrogen or oxygen intermediate conversion on metallic sites, thereby reducing competition between elementary steps.^37^ This spatial separation of functionality opens reaction pathways that are inaccessible on uniform, single-element surfaces.^40^

Figure 2 illustrates representative synergistic interfaces in multicomponent catalysts that exemplify the cooperative mechanisms relevant to HEA electrocatalysis.^23^ In Figure 2a, the Pt–Ni­(OH)_2_ interface establishes a spatially resolved HER pathway in which distinct reaction steps are distributed across chemically complementary sites. Ni­(OH)_2_ domains, owing to their oxophilic character, lower the kinetic barrier for water dissociation and supply protons via the Volmer step, while adjacent Pt sites efficiently catalyze proton reduction through the Heyrovsky step.^37,41^ This division of catalytic roles suppresses kinetic bottlenecks associated with single-site operation and highlights the advantage of spatially separated functionality.

**Figure 2:**
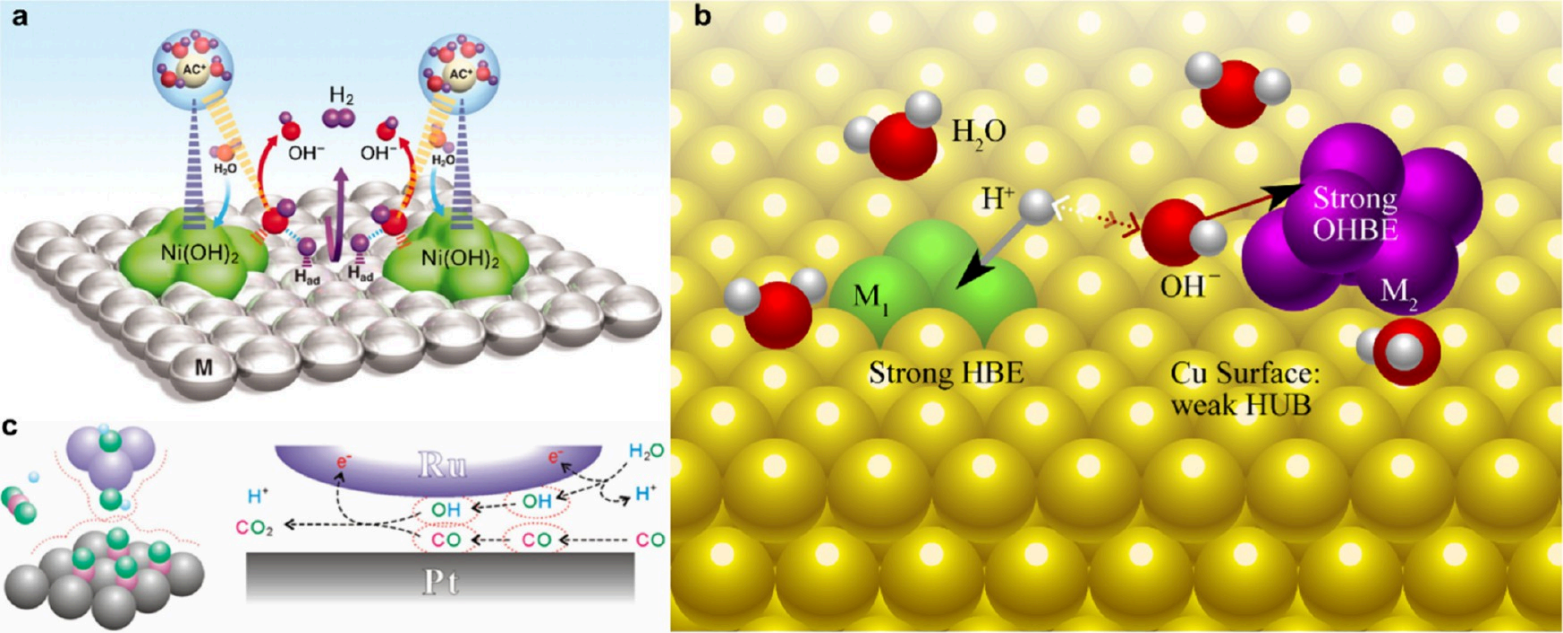
Representative synergistic interfaces in multicomponent catalysts illustrating cooperative reaction pathways. (a) A Pt–Ni­(OH)_2_ interface enables spatially separated HER steps, where Ni­(OH)_2_ promotes water dissociation and supplies protons, while adjacent Pt sites catalyze proton reduction, resulting in an efficient cooperative HER mechanism. Reproduced from Subbaraman et al., *Science*,^41^ **334**, 1256–1260 (2011), AAAS.* (b) Dual-site surface architectures allow independent optimization of hydrogen-binding and hydroxyl-binding energies, alleviating conventional scaling constraints and enhancing alkaline ORR activity.^42^ (c) Ru–Pt interfacial synergy facilitates CO oxidation, in which Ru generates mobile OH_ads_ species that migrate to neighboring Pt sites to remove strongly bound CO, thereby mitigating catalyst poisoning and improving turnover. Reprinted with permission from ref 43. Copyright 2007, American Chemical Society.

Figure 2b presents a dual-site surface design in which hydrogen adsorption and hydroxyl adsorption are independently optimized on chemically distinct motifs.^42^ By decoupling hydrogen-binding energy (HBE) and hydroxyl-binding energy (OHBE), this architecture circumvents the conventional scaling relations that constrain monometallic catalysts in alkaline ORR.^42,44^ The resulting enhancement in proton-transfer kinetics and OH^–^ desorption underscores how engineered heterogeneity can expand the accessible activity landscape beyond that dictated by single-descriptor optimization.^45^

**In** Figure 2c, Ru–Pt synergy provides a mechanistic framework for mitigating surface poisoning during CO oxidation.^46^ While Pt binds CO strongly and is susceptible to deactivation, neighboring Ru sites promote water activation and generate mobile OH_ads_ adsorbed species.^47^ These OH_ads_ species migrate to adjacent Pt–CO sites, facilitating oxidative CO removal and regenerating active Pt surfaces.^43,47,48^ This bifunctional interplay demonstrates how cooperative adsorption and intermediate transport between dissimilar elemental sites can stabilize catalytic activity under reactive conditions.^49^

Collectively, these examples illustrate how spatial, electronic, and chemical heterogeneity enable cooperative reaction pathways that are inaccessible on uniform surfaces. Such principles directly motivate the design of HEAs, where intrinsic atomic-scale disorder and compositional diversity naturally generate analogous synergistic motifs for HER, ORR, and related electrochemical processes.

## 3 Bifunctional Catalysts of HEAs for HER and ORR

### 3.1 Mechanistic Considerations of HER and ORR

As illustrated in Figure 3a, conventional electrocatalysts are typically optimized for a single dominant reaction pathway.^50,51^ In acidic electrolytes, HER proceeds via proton adsorption and electron transfer in the Volmer step, followed by molecular hydrogen formation through either the Tafel or Heyrovsky step.^52,53^ In alkaline environments, HER requires the initial activation of water molecules to generate adsorbed hydrogen, introducing an additional elementary step that is highly sensitive to surface composition and local coordination.^54,55^

**Figure 3:**
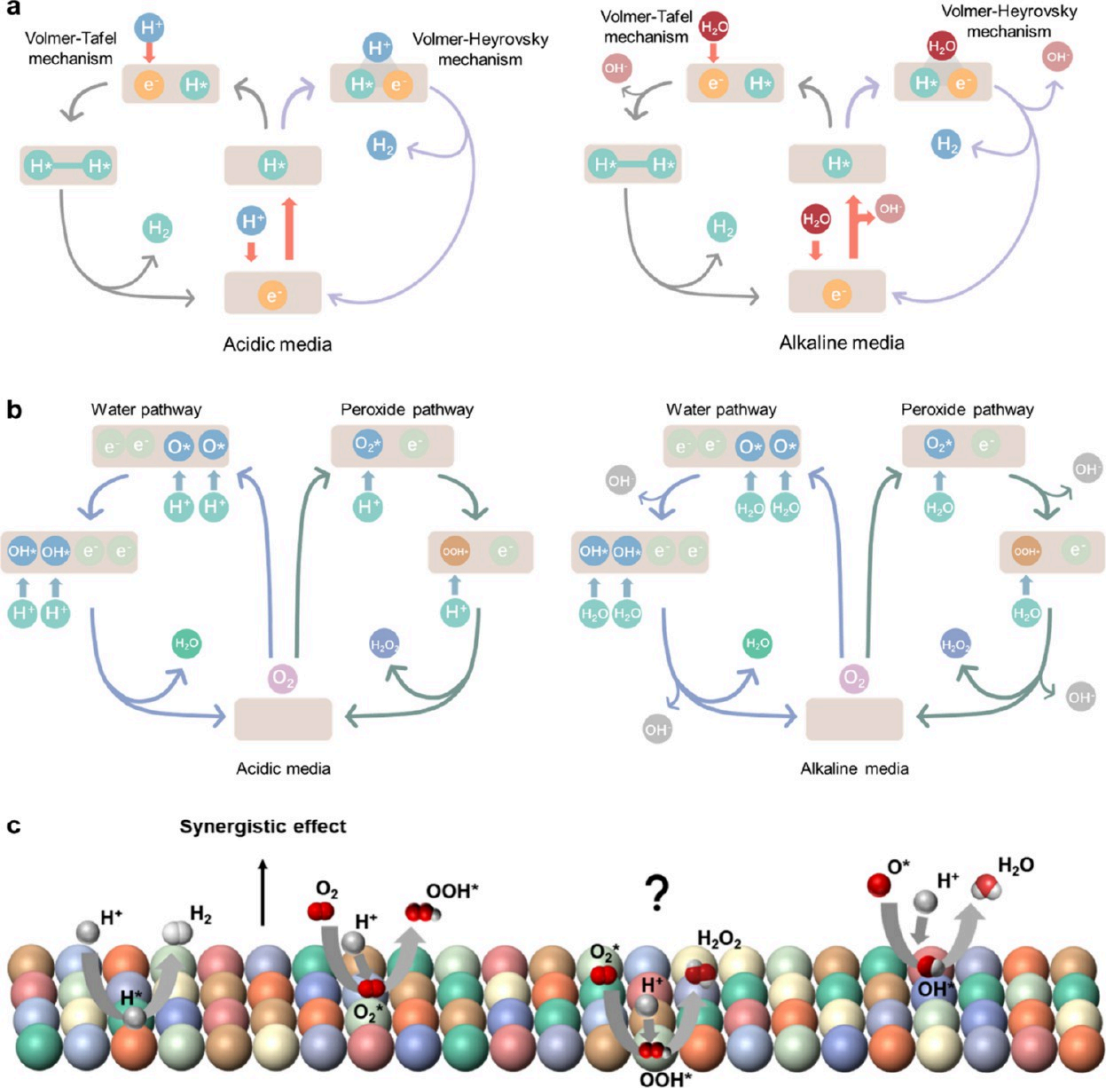
Mechanistic pathways of the HER and ORR in acidic and alkaline media, and the synergistic catalytic behavior enabled by HEAs. (a) HER mechanisms under acidic and alkaline conditions, highlighting the Volmer, Tafel, and Heyrovsky steps and their differing proton- or water-activation requirements. (b) ORR pathways, including the four-electron pathway leading to water (or OH^–^ in alkaline media) and the two-electron pathway producing peroxide intermediates, governed by the binding energetics of *O_2_, *OOH, *O, and *OH species. (c) Synergistic catalysis on HEA surfaces, where diverse local atomic environments provide multiple active sites that cooperatively facilitate proton adsorption, water dissociation, O_2_ activation, and *OOH conversion, thereby alleviating the bifunctional trade-offs inherent to monometallic catalysts.

Figure 3 summarizes the mechanistic pathways of HER and ORR under acidic and alkaline conditions, as well as the synergistic catalytic behavior enabled by HEAs.^56,57^ Figure 3b depicts the two primary ORR pathways: a four-electron pathway that directly reduces O_2_ to H_2_O in acidic media or OH^–^ in alkaline media, and a two-electron pathway that yields peroxide intermediates.^9,11^ The preferred reaction route and overall kinetics are governed by the adsorption energetics of oxygenated intermediates, including *O_2_, *OOH, *O, and *OH.^58^ While the four-electron pathway is generally favored in acidic electrolytes, both pathways may coexist in alkaline conditions depending on surface chemistry and local electronic structure. The challenge in designing bifunctional electrocatalysts arises from the fundamentally different adsorption requirements of HER and ORR.^59^ HER favors near-thermoneutral hydrogen adsorption, whereas ORR requires moderate binding of oxygenated intermediates to balance activation and desorption steps. These competing energetic requirements impose a well-recognized trade-off in conventional catalysts, making it difficult to simultaneously optimize both reactions on uniform surfaces.^60,61^

In contrast, Figure 3c illustrates how HEAs can mitigate this limitation through intrinsic compositional and structural heterogeneity.^3^ Multielement HEA surfaces naturally provide spatially and electronically distinct active sites that can preferentially facilitate different elementary steps. Certain local environments may promote HER-related processes such as water activation or proton adsorption,^62^ while others are better suited for ORR steps, including O_2_ activation and *OOH transformation.^9,63^ This distribution of catalytic roles emerges from the interplay between local atomic configuration and element-specific electronic structure.^56^

Beyond site diversity, the characteristic HEA effects, including lattice distortion, sluggish diffusion, high-entropy stabilization, and the cocktail effect,^22^ further modify coordination environments, adsorption energetics, and surface energy landscapes. As a result, reaction mechanisms on HEAs are inherently more complex and flexible than those on conventional catalysts.^64^ Although a complete mechanistic description of bifunctional catalysis on HEAs remains an open challenge, their compositionally diverse and dynamically responsive surfaces provide a robust materials basis for enabling concurrent HER and ORR activity.^3^

### 3.2 Bifunctional HEA Catalysts for HER and ORR

Recent advances demonstrate that rationally designed high-entropy alloy nanoparticles (HEA-NPs) can exhibit robust bifunctional electrocatalytic activity toward both HER and ORR. The combination of multicomponent synergy, tunable electronic structure, and nanoscale structural control enables the emergence of multiple active sites capable of catalyzing distinct reaction steps within a single material.

Figure 4 summarizes representative examples of HEA-NPs synthesized using different strategies. Figure 4a,b show size-tunable PtRuPdCoNi HEA nanoparticles prepared via a confined-space synthesis method. Decreasing particle size leads to pronounced changes in polarization behavior, reflecting enhanced activity for both HER and ORR. Complementary density functional theory (DFT) calculations indicate that smaller particles exhibit a downshifted d-band center, resulting in more favorable hydrogen adsorption free energies (Δ*G*_H*_) and reduced energetic barriers for *OOH formation, both of which contribute to improved bifunctional performance.^25^

**Figure 4:**
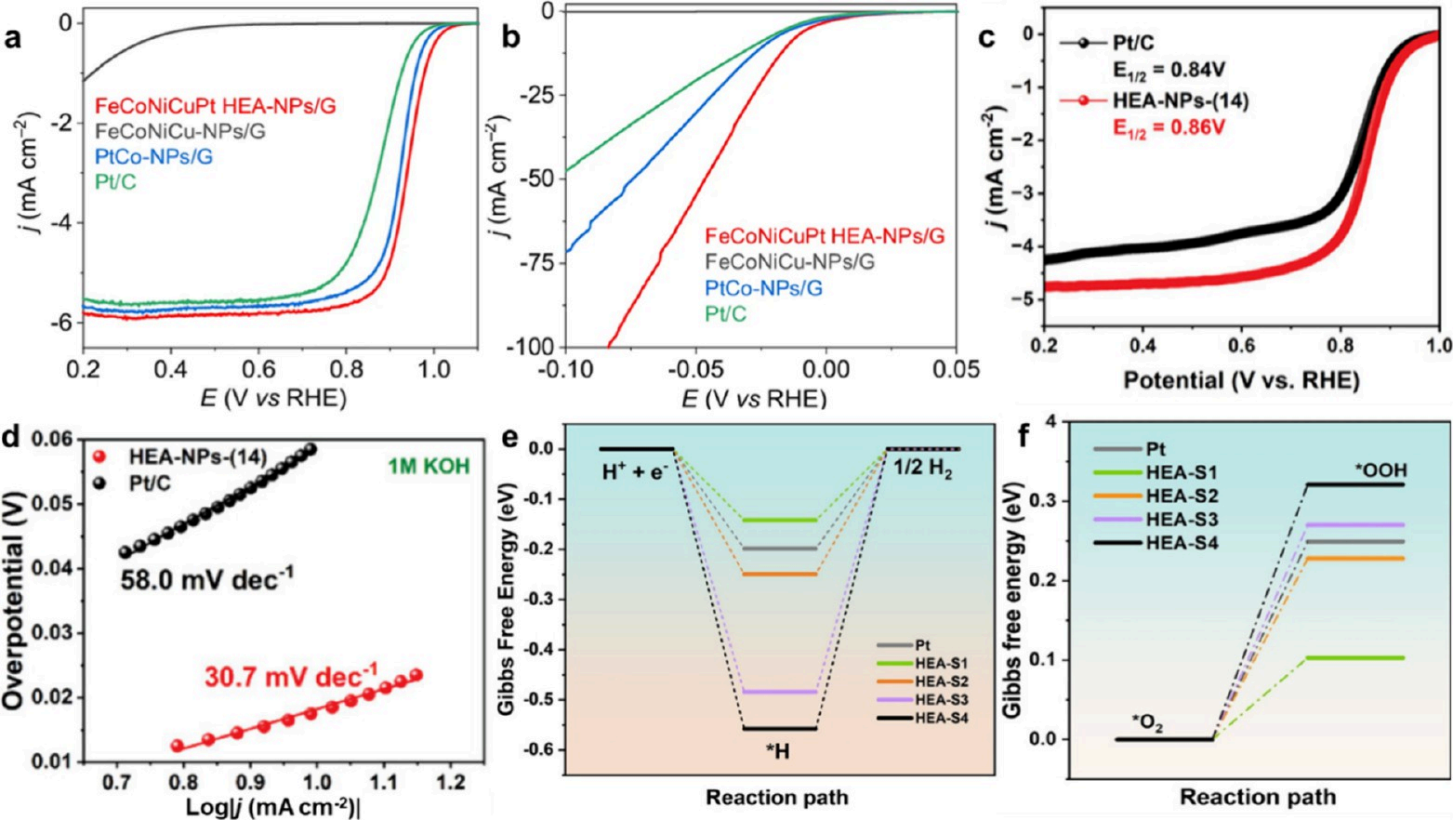
Bifunctional electrocatalytic performance of High Entropy Alloy-Nanoparticles (HEA-NPs) prepared via different synthetic strategies. (a and b) Size-tunable PtRuPdCoNi HEA nanoparticles synthesized through a confined space method. Smaller particles reveal a more downshifted d band center along with more optimal Δ*G*_H*_ and better energetics for *OOH formation, which together lead to enhanced HER and ORR activity.^25^ Reproduced with permission from ref 25. Available under a CC-BY 4.0 license. Copyright 2025 Springer Nature. (c and d) Multielement HEA nanoparticles with up to 14 elements obtained by a step alloying strategy. These catalysts exhibit exceptional bifunctionality, reaching an ORR half-wave potential of 0.86 V versus Reversible Hydrogen Electrode (RHE) and a Tafel slope of 30.7 mV dec^–1^ for HER, which is indicative of very fast charge transfer kinetics.^26^ Reprinted with permission from ref 26. Copyright © 2023 Wiley. (e and f) FeCoNiCuPt HEA nanoparticles made by hydrothermal flow synthesis and supported on carbon. These compounds produce more positive ORR half-wave potentials than commercial Pt/C and have lower overpotentials for HER, thus confirming accelerated reaction kinetics.^24^ Reproduced with permission from ref 24. Copyright©YYYY John Wiley & Sons.

Using a different design approach, Wang et al. employed a step-alloying strategy to synthesize multielement HEA nanoparticles containing up to 14 constituent elements while maintaining a homogeneous solid-solution structure. As shown in Figure 4c,d, these High entropy alloy-Nanoparticles (HEA-NPs) exhibit an ORR half-wave potential of 0.86 V versus Reversible Hydrogen Electrode (RHE), exceeding that of commercial Pt/C, along with a low HER Tafel slope of 30.7 mV dec^–1^. These metrics indicate efficient charge transfer and hydrogen binding strengths close to optimal values. The reported electrochemical performance highlights the effectiveness of extensive compositional tuning in modulating electronic structure and catalytic behavior.^26^

In a third approach, FeCoNiCuPt HEA nanoparticles were synthesized via hydrothermal flow methods and uniformly supported on carbon substrates. Figure 4e,f show that these catalysts deliver enhanced ORR activity relative to Pt/C, as evidenced by more positive half-wave potentials, together with reduced HER overpotentials and steeper polarization slopes. These results indicate accelerated reaction kinetics and improved adsorption energetics, demonstrating the viability of scalable synthesis routes for producing catalytically active HEA nanostructures.^24^ Collectively, these studies illustrate how compositional diversity, uniform atomic dispersion, and strain-induced electronic modulation in HEAs generate a broad spectrum of active sites, enabling efficient bifunctional catalysis across HER and ORR.^62^

Beyond catalytic activity, the long-term durability and structural stability of HEA-NPs are critical for practical electrochemical applications. Uniform elemental mapping by High-Angle Annular Dark-Field Scanning Transmission Electron Microscopy (HAADF-STEM) (Figure 5a) demonstrates that each nanoparticle contains all five constituent elements in a homogeneous manner, confirming the formation of a multicomponent solid-solution phase. This uniform elemental distribution, together with the pronounced lattice distortion revealed by clear lattice fringes and Selected Area Electron Diffraction (SAED) patterns indexed to the (111) and (200) planes in Figure 5b, provides direct evidence of the single-phase nature and intrinsic structural robustness of the HEA framework.^26^

**Figure 5:**
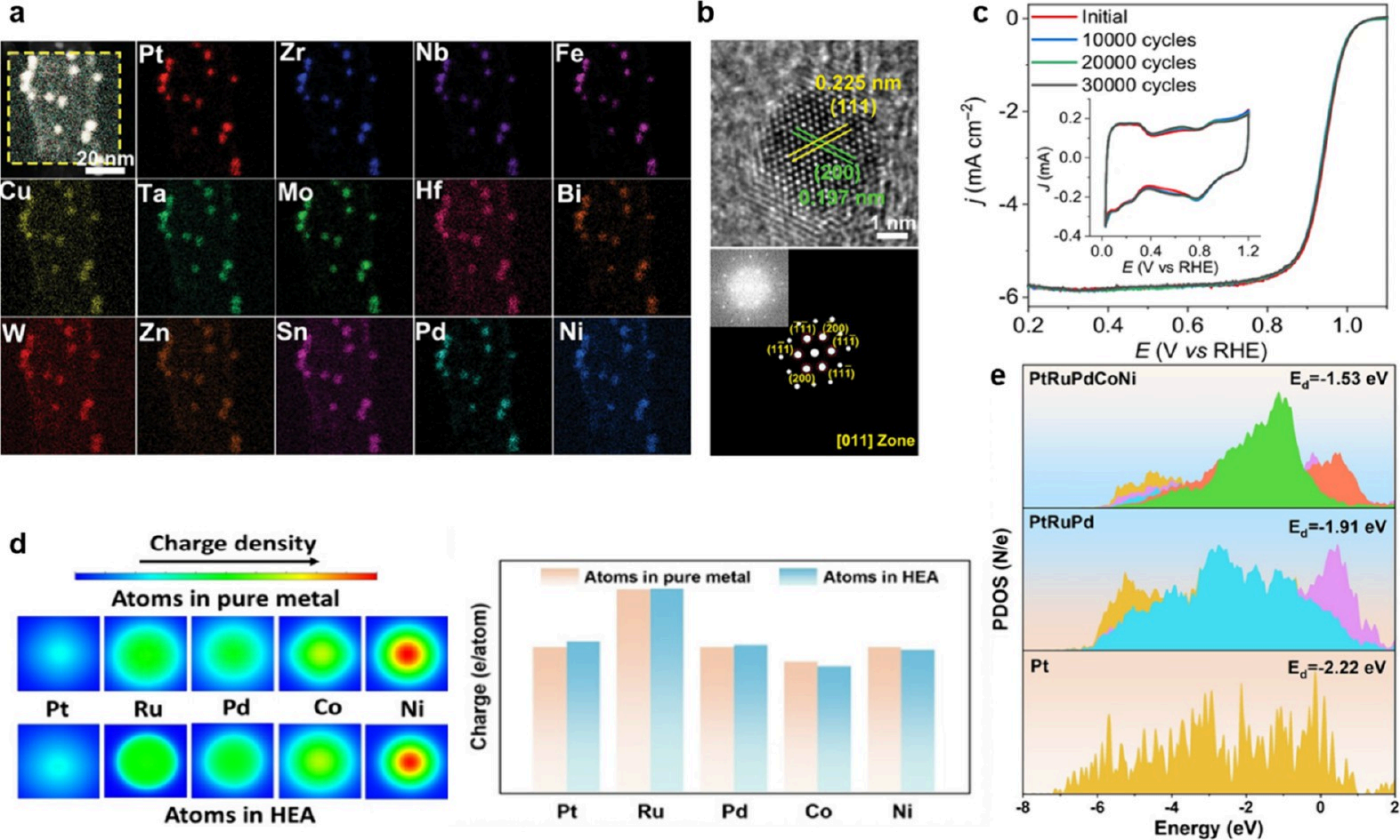
Structural durability and electronic stabilization of HEA-NPs. (a and b) High-Angle Annular Dark-Field Scanning Transmission Electron Microscopy (HAADF-STEM) elemental mapping and atomic-resolution imaging reveal homogeneous multimetal distribution, single-phase crystallinity, and pronounced lattice distortion, with lattice fringes and Selected Area Electron Diffraction (SAED) patterns confirming structural integrity.^26^ Reproduced with permission from ref 26. Copyright © 2023 John Wiley & Sons. (c) PtRuPdCoNi HEA-NPs exhibit excellent electrochemical stability, retaining over 90% of the initial current density after 30,000 cycles, attributed to suppressed atomic diffusion and inhibited nanoparticle coalescence.^25^ Reproduced with permission from ref 25. Available under a CC-BY 4.0 license. Copyright 2025 Springer Nature. (d and e) Charge density difference and projected density of states (PDOS) analyses demonstrate substantial interelement electronic redistribution and an upward shift of the Pt d-band center relative to monometallic Pt, leading to moderated intermediate adsorption and enhanced resistance to surface poisoning.^24^ Reproduced with permission from ref 24. Copyright © 2025 John Wiley & Sons.

This structural integrity directly translates into electrochemical durability. As shown in Figure 5c, ultrafine PtRuPdCoNi HEA-NPs retain more than 90% of their initial current density after 30,000 electrochemical cycles, with negligible changes in polarization behavior. Such remarkable stability is attributed to sluggish atomic diffusion and strong lattice distortion within the multicomponent matrix, which collectively suppress nanoparticle coalescence and surface reconstruction during prolonged cycling.^25^

In addition to structural robustness, HEAs exhibit tunable electronic properties that further enhance surface stability. Figure 5d reveals substantial charge redistribution among Pt, Ru, Pd, Co, and Ni relative to their monometallic counterparts, indicating strong interelement electronic interactions and extensive charge delocalization. Consistent with this observation, the projected density of states (PDOS) in Figure 5e shows an upward shift of the Pt d-band center, leading to moderated adsorption of reaction intermediates and reduced susceptibility to surface poisoning. Taken together, the combined structural and electronic stabilization mechanisms indicate that HEA nanoparticles are inherently resistant to corrosion, aggregation, and phase segregation. The synergy of these attributes positions HEAs as a promising class of bifunctional electrocatalysts with the durability and efficiency required for long-term energy conversion applications.^24^

The mechanistic analysis above indicates that bifunctional activity in HEAs arises from compositionally and structurally heterogeneous active sites rather than from a single optimal motif. Achieving this level of complexity in real materials therefore requires advanced synthetic strategies capable of precisely controlling elemental distribution, particle size, and interfacial structure. These challenges are discussed in the following section.

## 4 Synthesis of HEAs for HER and ORR

Early studies of HEAs primarily focused on bulk materials synthesized using conventional metallurgical techniques, such as arc melting, induction melting, melt spinning, mechanical alloying, laser ablation, high-energy ball milling, and spark plasma sintering.^65–69^ These approaches typically involve high-temperature melting followed by rapid solidification to promote elemental mixing and the formation of single-phase solid solutions, resulting in alloys with face-centered cubic (FCC), body-centered cubic (BCC), or hexagonal close-packed (HCP) crystal structures.^70,71^

While effective for producing bulk HEAs, these methods are not well suited for electrocatalytic applications, which require high surface area, nanoscale particle sizes, tunable surface structures, and compatibility with conductive supports.^70,72^ Bulk HEAs generally suffer from low active-site utilization, limited intrinsic activity, and insufficient interface engineering, all of which constrain their performance in bifunctional electrocatalysis.^73^ Consequently, increasing attention has been directed toward the synthesis of HEA-NPs, where multicomponent synergistic effects and a high density of accessible surface sites can be more effectively exploited.^70^

Meeting these requirements necessitates the development of advanced synthetic strategies capable of fully leveraging the compositional complexity and catalytic tunability of HEA-NPs. Given the nearly limitless combinations of constituent elements and the associated atomic-scale complexity, precise control over particle size, elemental distribution, and dispersion on conductive supports is essential for achieving optimal electrocatalytic performance.^74^ In response, a range of scalable and robust synthesis methods has been developed, offering diverse routes to the rational design of bifunctional HEA catalysts.^74,75^

This section is not intended to provide an exhaustive survey of all reported synthesis strategies. Instead, representative case studies are selected to distill general design principles and practical considerations relevant to bifunctional HEA electrocatalysts. In Figure 6a, electrospinning-assisted metal–organic frameworks (MOF) confinement is employed, where multimetal precursors encapsulated within MOFs are combined with polyacrylonitrile (PAN) and electrospun into composite fibers. Subsequent pyrolysis converts PAN into conductive carbon and decomposes the MOF, yielding well-dispersed HEA-NPs embedded within porous carbon nanofibers. The combined confinement effects of the MOF and fibrous carbon matrix effectively regulate nanoparticle nucleation and suppress aggregation at elevated temperatures, while the resulting fiber morphology facilitates mass transport and exposes abundant active sites.^24^

**Figure 6:**
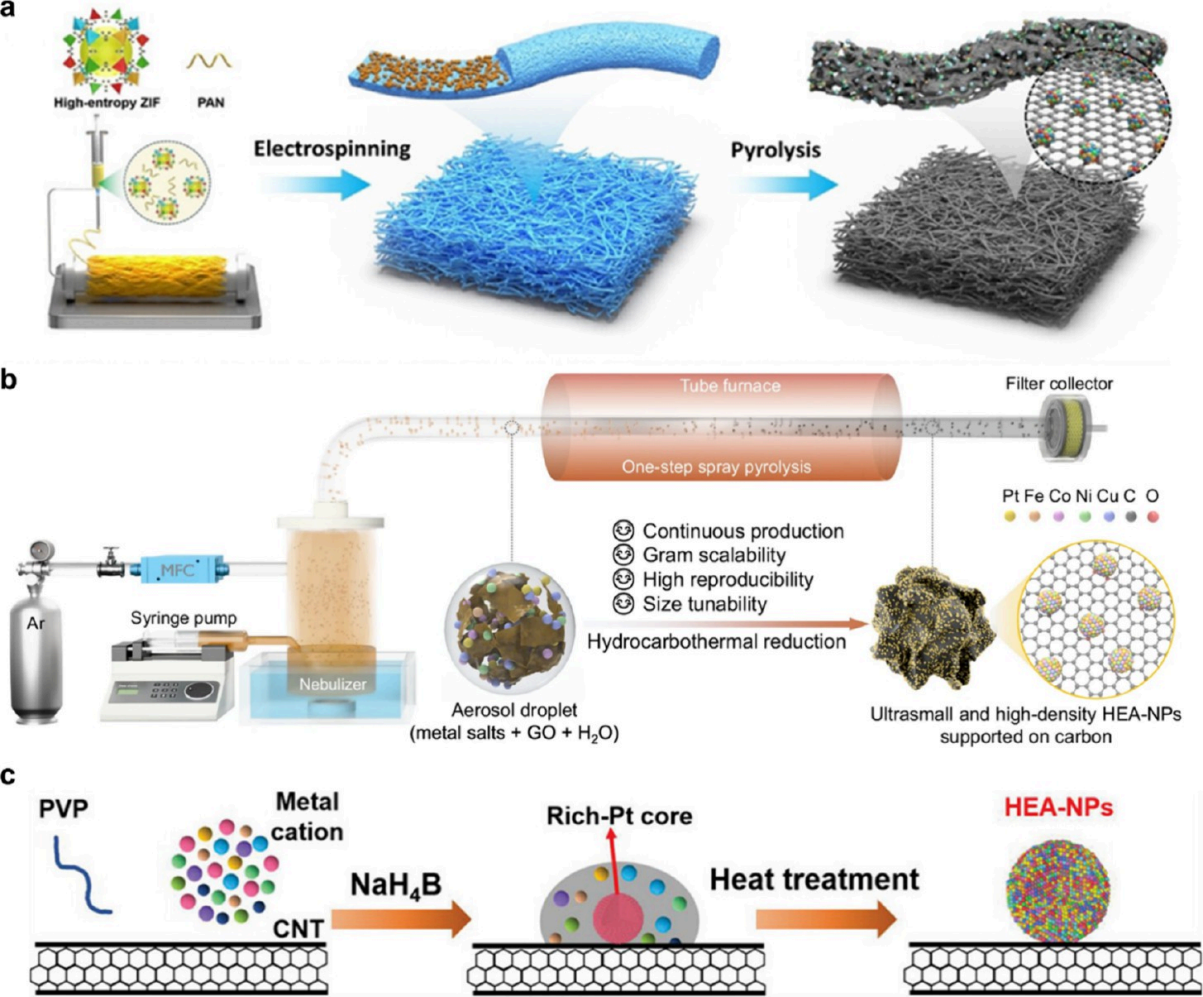
Schematic illustrations of representative synthetic strategies for HEA-NPs. (a) Electrospinning-assisted metal–organic frameworks (MOFs) confinement, in which multimetal precursors encapsulated within MOFs and polyacrylonitrile (PAN) fibers are pyrolyzed to yield well-dispersed HEA-NPs embedded in porous carbon nanofibers.^24^ Reproduced with permission from ref 24. Copyright © 2025 John Wiley & Sons. (b) Continuous-flow spray pyrolysis, where aerosolized solutions of metal salts and graphene oxide undergo rapid thermal decomposition to produce ultrasmall, uniformly distributed HEA-NPs on carbon supports.^25^ Reproduced with permission from ref 25. Available under a CC-BY 4.0 license. Copyright 2025 Springer Nature. (c) Step-alloying synthesis involving sequential ion anchoring, NaBH_4_ reduction to form a Pt-rich core, and subsequent thermal diffusion to generate compositionally homogeneous HEA-NPs.^26^ Reproduced with permission from ref 26. Copyright © 2023 John Wiley & Sons.

Among the reported methods, continuous-flow spray pyrolysis (CFSP), shown in Figure 6b, is particularly notable for its scalability and ability to control nanoparticle dispersion. In this process, ultrasonic nebulization generates aerosol droplets containing metal salts and graphene oxide dispersed in water, which are subsequently transported into a heated tubular reactor. Rapid thermal decomposition, accompanied by in situ hydrocarbothermal reduction driven by hydrogen generated from carbon–water reactions, promotes uniform alloy formation without the need for external reducing agents. The short residence time and rapid heating rates effectively limit particle coalescence, producing ultrasmall, single-phase HEA-NPs suitable for large-scale production.^25^

Figure 6c depicts a modular step-alloying strategy that enables fine control over composition and elemental distribution. In this approach, metal cations are sequentially anchored onto polyvinylpyrrolidone (PVP)-stabilized carbon nanotubes, followed by chemical reduction to form a Pt-rich metallic core. Subsequent thermal treatment drives atomic diffusion and alloying of additional elements, resulting in compositionally homogeneous HEA-NPs containing up to 14 constituent elements. This stepwise method provides exceptional control over compositional complexity and electronic structure, both of which are critical parameters for tailoring catalytic behavior. Therefore, the synthesis strategies mentioned above offer complementary pathways toward achieving precise structural control, compositional homogeneity, and scalability. By integrating atomic-level design with tunable processing conditions, these approaches constitute a versatile toolkit for the development of next-generation HEA-based bifunctional electrocatalysts for HER, ORR, and broader energy conversion applications.^26^

Although advanced synthetic strategies have significantly improved control over composition, particle size, and elemental distribution in HEA-based electrocatalysts, the resulting materials still exhibit substantial atomic-scale complexity and heterogeneity. This intrinsic disorder makes it challenging to establish unambiguous structure–activity relationships across the vast compositional and configurational space using experimental approaches alone. Consequently, computational and data-driven methods have increasingly been integrated with experiments to address this limitation. These approaches enable systematic interrogation of diverse local atomic environments, thereby facilitating rational catalyst design, as discussed in the following section.

## 5 Computational Design of HEAs for Bifunctional Electrocatalysts

Despite their demonstrated potential, the experimental discovery and optimization of HEA electrocatalysts face several inherent challenges. The enormous configurational space arising from the large number of possible elemental combinations makes comprehensive experimental exploration through trial-and-error approaches impractical.^76,77^ In addition, precursor availability and synthesis costs can impose significant constraints, particularly when noble metals or complex multistep synthetic routes are required. Moreover, accurate characterization of atomic-scale elemental distributions and surface compositions remains difficult, and experimentally synthesized HEAs often exhibit structural and compositional heterogeneity.^78^ This heterogeneity complicates the establishment of clear structure–activity relationships.^79^ Finally, isolating the influence of local geometric and electronic environments on catalytic performance is challenging using experimental techniques alone, especially under realistic electrochemical operating conditions where surface reconstruction and dynamic restructuring occur.^20,69^ Together, these limitations highlight the need for approaches that can systematically probe compositional complexity, resolve local atomic environments, and disentangle structure–property relationships beyond the reach of experiment alone.

Within this context, computational methodologies have emerged as indispensable tools for the design and understanding of HEA electrocatalysts. Techniques such as density functional theory (DFT), atomistic modeling, and machine learning (ML) enable systematic exploration of vast compositional and configurational spaces that are inaccessible to experiments. By providing atomic-level insight into local geometric and electronic environments, these methods facilitate the establishment of structure–activity relationships, support high-throughput screening and rational catalyst design, and significantly accelerate materials discovery while reducing reliance on costly and time-consuming experimental trial-and-error.

### 5.1 Modeling Approaches for Complex HEA Structures

Accurately depicting the intrinsic disorder of HEAs is the basis for knowing their thermodynamic stability, electronic structure, mechanical response, and catalytic behavior. As a result of their multicomponent composition, highly distorted lattice, and totally random atomic arrangements, HEAs present a great challenge to existing alloy modeling frameworks.^78^ In the last decades, numerous theoretical strategies have been introduced to compromise between accuracy and computational feasibility, namely mean field approximations, quasi-random supercells, and statistical sampling approaches.^80^ Figure 7 shows the representative structural models for these different approaches.

**Figure 7:**
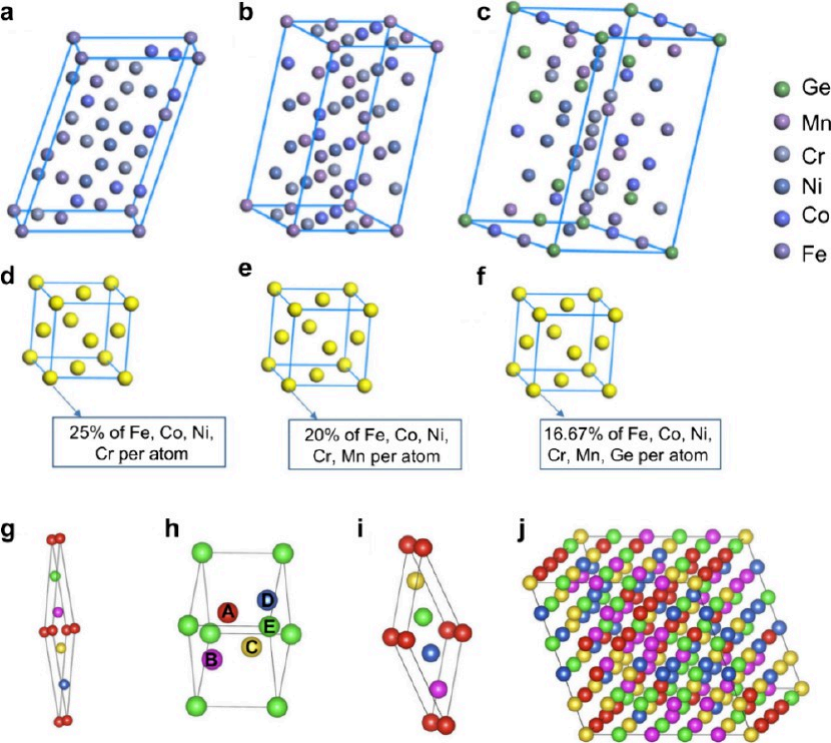
Representative structural modeling strategies for capturing chemical disorder in HEAs. (a–c) Special quasirandom structure (SQS) models for FeCoNiCr-based alloys, reproducing near-neighbor correlation functions and lattice distortions characteristic of random multicomponent solid solutions.^81^ Reproduced with permission from ref 81. Copyright © 2021 Elsevier. (d–f) Virtual crystal approximation (VCA) models for the same systems, in which each lattice site is represented by an averaged virtual atom; VCA provides reasonable agreement with SQS when constituent elements have similar atomic sizes and electronic structures, but fails in the presence of significant chemical or size mismatch (e.g., Ge-containing alloys).^81^ Reproduced with permission from ref 81. Copyright © 2021 Elsevier. (g–i) Small set of ordered structures (SSOS), consisting of three minimal ordered configurations whose weighted ensemble reproduces short-range statistical features of random alloys at substantially reduced computational cost compared with SQS.^82^ Reprinted with permission from ref 82. Copyright © 2016 American Physical Society. (l) Representative fully random multicomponent configuration illustrating the diverse local atomic environments that realistic HEA models must capture.^82^ Reprinted with permission from ref 82. Copyright © 2016 American Physical Society.

#### 5.1.1 Mean-Field Approaches

Mean field methods like the virtual crystal approximation (VCA) and the coherent potential approximation (CPA) treat chemical disorder by means of replacing a heterogeneous lattice with an effective medium.^83–85^ In VCA, each lattice site gets a single “virtual atom” whose potential stands for a weighted average of the constituent elements.^86^ CPA goes beyond that by adding some corrections for electron scattering arising from the fluctuations of atomic potentials.^87^ Both approaches provide computationally efficient access to macroscopic properties, including equilibrium lattice parameters and mean electronic structures.

However, these approaches inherently average over local atomic environments and therefore fail to capture local lattice distortions and short-range chemical fluctuations, which are central to the structure of high-entropy alloys. This limitation becomes evident when comparing VCA representations with corresponding special quasirandom structure (SQS) models.^88^ For example, in Figure 7a,d, metal alloys made up of chemically similar 3d transition metals only, such as Fe, Co, Ni, Cr systems, show almost perfect cubic symmetries both in VCA and SQS,^81^ and the same degree of conformity is present in the five-component FeCoNiCrMn mixture depicted in Figure 7b,e.^81^ In contrast, pronounced deviations arise when one of the alloying elements exhibits a significantly larger atomic radius and a distinct electronic configuration. This behavior arises because the SQS model of the FeCoNiCrMnGe alloy exhibits significant lattice distortion, reflecting the local structural response to atomic size and chemical mismatch (Figure 7c,f). In contrast, the corresponding VCA representation remains artificially cubic, an artifact of the averaged potential inherent to the method.

#### 5.1.2 Special Quasirandom Structures (SQS)

The SQS approach provides an explicit atomistic representation of chemical disorder by constructing a finite periodic supercell whose near-neighbor correlation functions closely match those of an ideal random alloy. In practice, SQS models are generated by optimizing atomic arrangements so that pair (and sometimes higher-order) correlation functions reproduce those expected for a perfectly random solid solution.^88,89^ For complex quaternary and quinary HEAs, SQS structures can be efficiently generated using tools such as Alloy Theoretic Automated Toolkit (ATAT), and numerous studies have shown that this approach reliably captures key bulk properties, including formation energies, elastic constants, phonon spectra, and local lattice distortions.^90,91^

Figure 7j shows a representative 125-atom SQS supercell for a quinary bcc alloy. Such large supercells are typically required to accurately reproduce first- and second-nearest-neighbor correlations in random multicomponent systems.^82,92^ While this explicit treatment of disorder enables high fidelity in describing local structural relaxation and electronic structure, it also incurs substantial computational cost, particularly for first-principles calculations involving large compositional spaces or surface models. Moreover, an individual SQS represents only a single realization of disorder and therefore does not explicitly sample the full statistical distribution of local atomic environments.^5^ In particular, for surface catalysis studies, a single SQS slab cannot represent the full adsorption energy distribution arising from diverse local atomic motifs. This limitation is especially relevant for catalytic HEAs, where variability in adsorption energies arises from a broad ensemble of distinct surface sites rather than a single structural configuration.

#### 5.1.3 Small Set of Ordered Structures (SSOS)

To mitigate the sizable computational demand of SQS as well as the absence of local environment resolution in mean field methods, the Small Set of Ordered Structures (SSOS) method was developed as a resourceful statistical framework for modeling random multicomponent solid solutions.^82,93^ The principal idea of SSOS is that three five-atom structures, each with a different atomic ordering, representing a quinary alloy, are shown in Figure 7g–i. The combination of the three is intended to reproduce the first and second nearest neighbor correlation functions of a 125-atom SQS depicted in Figure 7j, even though each of the structures is extremely small. In fact, energies and volumes averaged over the ensemble and derived from these small SSOS models are within a few meV per atom of SQS results, thus the computational cost is lowered by more than 1 order of magnitude.^82^

Moreover, the sampling method inspired by SSOS has been extensively used in HEA surface modeling besides bulk systems, where catalytic behavior is the result of diverse local atomic configurations. By preparing a representative ensemble of small slab models with different surface site occupations, SSOS makes the adsorption energy distributions an efficient estimation and the identification of active motifs possible, i.e., functionalities that are exceptionally difficult to realize by a single SQS surface model.

#### 5.1.4 Machine Learning-Driven Generative Models

Recently, the data-driven materials design has been revolutionized by the generative ML models that are used for quickly and efficiently exploring the vast compositional and structural spaces of HEAs. Such models, including variational autoencoders (VAEs), graph neural networks (GNNs), and diffusion-based models, are capable of learning the latent structure property relationships from the existing density functional theory (DFT) data sets and, hence, can suggest new stable HEA configurations having the desired properties.

Li et al. used a generative model informed by physics that was based on a diffusion variational autoencoder framework. Besides that, they have introduced short-range order constraints and particle swarm optimization iterations to obtain stable structure modeling of multicomponent amorphous materials.^94^ The resulting structures were confirmed through theory and experiment to exhibit excellent performance in OER. Complementing such atomic-level structural modeling, generative architectures have also been extensively deployed for the compositional design of HEAs. For instance, Debnath et al. utilized Conditional Generative Adversarial Networks (CGAN) to model the complex mapping between latent compositional spaces and mechanical properties, facilitating the inverse design of refractory HEAs.^95^ Furthermore, extending the modeling scope to property-driven discovery, Rao et al. integrated generative models with Markov Chain Monte Carlo (MCMC) sampling to efficiently traverse the HEA space, successfully identifying candidates with targeted thermal expansion coefficients.^96^ These diverse approaches collectively advance the generative modeling of HEAs from macroscopic composition screening to microscopic atomic ordering.

From a practical modeling standpoint, these approaches occupy complementary roles in balancing accuracy, computational cost, and physical fidelity when describing disordered HEA structures. Mean-field methods, such as VCA and CPA, provide high computational efficiency and are well suited for rapid screening of bulk thermodynamic and electronic trends; however, by construction, they average over local atomic environments and therefore cannot capture lattice distortions or short-range chemical fluctuations. In contrast, SQS models offer explicit atomistic representations of chemical disorder and reproduce near-neighbor correlation functions, enabling more accurate descriptions of local structure and energetics, albeit at significantly higher computational cost and with limited statistical sampling. Local site-sampling strategies, including SSOS and related ensemble-based approaches, further bridge this gap by approximating disorder statistics through a reduced set of representative configurations, thereby achieving a favorable trade-off between accuracy and efficiency. Such methods are particularly advantageous for modeling catalytic surfaces, where activity emerges from a distribution of distinct local environments rather than a single idealized structure. Collectively, these frameworks constitute a hierarchical modeling toolkit, allowing the level of complexity to be matched to the target properties and available computational resources.

Nevertheless, as the compositional and configurational dimensionality of HEAs expands, even ensemble-based atomistic approaches struggle to efficiently sample the vast space of local environments and to uncover emergent structure–property relationships. This limitation has driven the growing integration of machine learning–based generative models, which provide a complementary strategy for learning latent representations of disorder and accelerating exploration of complex HEA design spaces.

### 5.2 Mechanism Elucidation and Performance Evaluation

#### 5.2.1 Mechanism Elucidation of the HEA Catalyst

A mechanistic understanding of catalytic processes is fundamental to the rational design and performance optimization of electrocatalysts. For conventional catalyst systems, reaction pathways can often be systematically analyzed using well-established computational frameworks. Descriptor-based approaches such as volcano plots, linear scaling relations, and structure–activity correlations are widely employed to connect surface electronic structure and adsorption energetics with catalytic activity and selectivity.^97–99^ These methodologies have played a central role in catalyst screening and materials development.

A representative example is the seminal work by No̷rskov and co-workers, who introduced the hydrogen adsorption free energy (Δ*G*_H*_) calculated from DFT as a single descriptor for evaluating HER activity across metal surfaces, leading to the widely adopted HER volcano relationship.^44^ Similarly, for ORR, the binding energies of oxygenated intermediates (*O, *OH, and *OOH) have been identified as key descriptors that govern reaction kinetics and pathway selection, enabling rational identification of active materials under different electrochemical conditions.^11^ In the context of single-atom catalysis, Wang and Chen et al. further demonstrated that the local coordination environment of isolated Fe centers in nitrogen-doped carbon can be systematically correlated with ORR activity through descriptors associated with charge transfer and spin state.^100^ These descriptor-based frameworks are effective largely because traditional catalyst systems typically feature relatively simple and uniform active-site geometries.^14^ Under such conditions, assumptions including site homogeneity and linear scaling relationships remain reasonably valid, allowing catalytic mechanisms to be simplified and interpreted with clarity. Consequently, computational predictions based on these descriptors often show good agreement with experimental observations, providing a reliable basis for rational catalyst design.

However, HEAs present substantially greater complexity. Their catalytic behavior is governed by a dramatic expansion in compositional diversity, local structural heterogeneity, and adsorption-site variability.^64,101–103^ This complexity significantly increases computational demands and complicates mechanistic interpretation, as catalytic performance arises from the coupled effects of electronic structure, local geometry, lattice strain, and elemental interactions. These factors are intrinsically intertwined and cannot be readily isolated. Moreover, HEA surfaces host a broad distribution of nonequivalent active sites rather than a single representative motif, and there is currently no unified theoretical framework capable of fully capturing this complexity.^64,104^ As a result, reliance on simple, single-value descriptors such as a characteristic ΔG_H*_ for HER or a single oxygen-intermediate binding energy for ORR, can lead to oversimplified interpretations. Although such descriptors may correlate with overall activity trends, they fail to resolve the site-specific contributions of different elements. This limitation obscures mechanistic insight and hinders interpretable, element-resolved catalyst design. To overcome the limitations of conventional descriptors, data-driven methodologies have emerged as powerful tools for extracting physically meaningful features from complex catalytic systems. Machine learning (ML), in particular, enables systematic exploration of the vast compositional and configurational spaces characteristic of HEAs while identifying interpretable descriptors linked to catalytic performance.^105,106^

As a representative example of interpretable, small-data modeling, the sure independence screening and sparsifying operator (SISSO) framework has been widely adopted. Recently, Oh et al. demonstrated that coupling SISSO with the agreement (α) method significantly enhances model robustness when dealing with sparse data sets.^106^ As shown in Figure 8a, the prediction performance for the conduction band minimum (ε_CBM_) is compared between the standard SISSO framework and the SISSO+α approach. While the standard SISSO model exhibits signs of overfitting, evidenced by the divergence of the validation RMSE (purple) from the training/testing scores, the SISSO+α model maintains superior adaptability and low error rates across all data sets. Notably, the validation set in this study was composed of quaternary compounds, whereas training was performed on simpler ternary systems. The ability of the SISSO+α model to generalize to these higher-order complexities is further highlighted by the inset parity plot in Figure 8a, where predictions remain tightly clustered around the diagonal even as the number of training points is drastically reduced. This demonstrates that advanced symbolic regression techniques can effectively identify physical descriptors that hold true across different regions of the chemical space.

**Figure 8:**
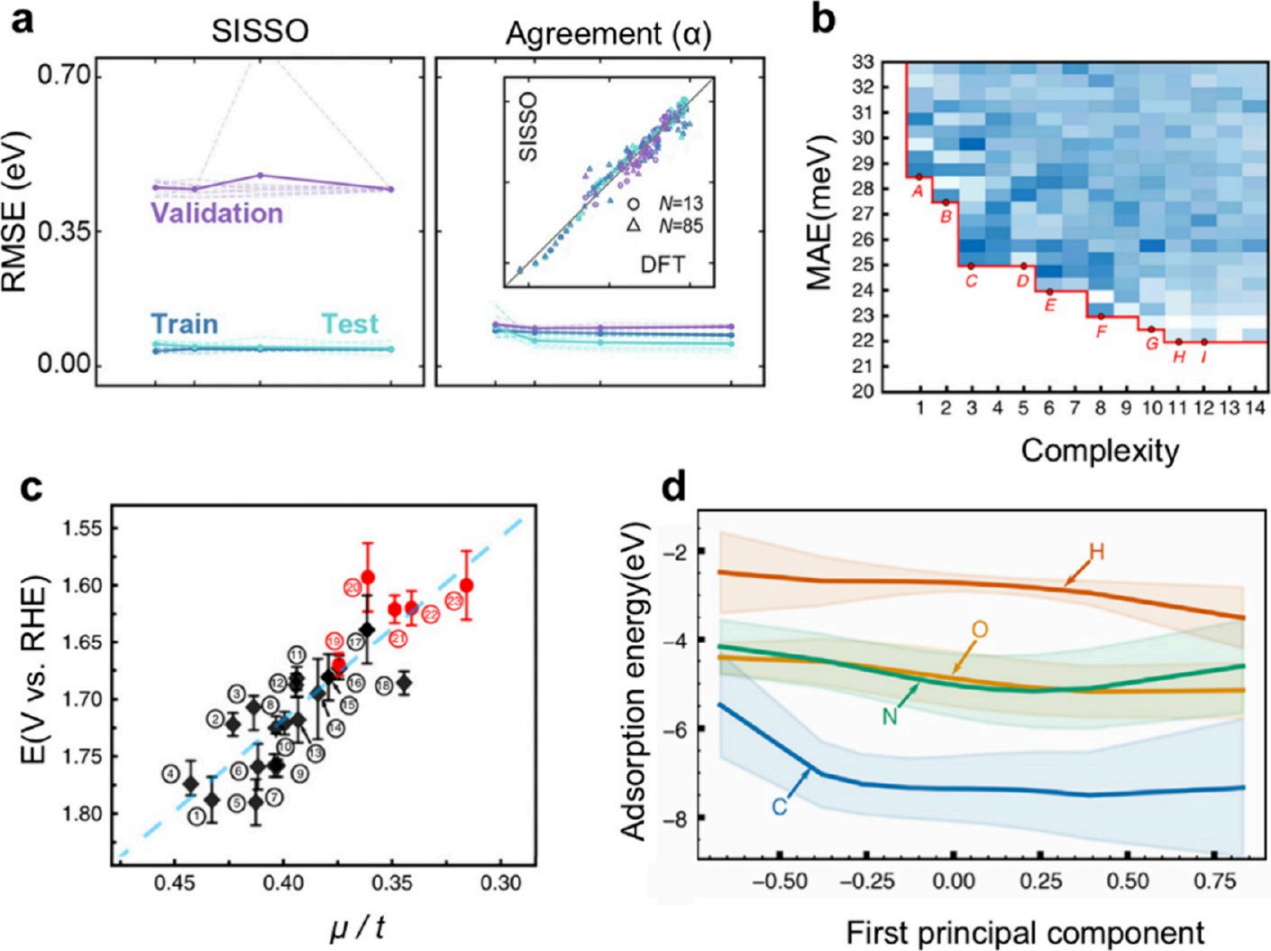
Computational elucidation of catalytic mechanisms using AI-driven feature discovery. (a) The SISSO+α method enables robust descriptor discovery from sparse data sets. Reproduced from ref 106. Available under a CC-BY 4.0 license. Copyright 2024 Springer Nature. (b) Symbolic regression (SR) analysis of perovskite oxides displays the Pareto front, which identifies optimal descriptors by balancing mathematical complexity with prediction error (MAE). Reproduced from ref 107. Available under a CC-BY 4.0 license. Copyright 2020 Springer Nature. (c) The SR-derived descriptor μ/*t* (octahedral factor divided by tolerance factor) exhibits a strong linear correlation with OER activity (*V*_RHE_), validating its use for rapid materials screening. Reproduced from ref 107. Available under a CC-BY 4.0 license. Copyright 2020 Springer Nature. (d) Partial dependence plots derived from unsupervised Principal Component Analysis (PCA) reveal that the first principal component (PC1) of the electronic density of states effectively governs the adsorption energy trends for C, O, N, and H species. Reproduced with permission from ref 108. Copyright © 2021 Elsevier.

In addition to directly extracting critical features, machine learning also serves as a powerful tool for discovering novel, high-value descriptors that capture complex local interactions. Yang et al. combined 3D atomic electron tomography (AET) with DFT to formulate a machine-learning-derived Local Environment Descriptor (LED).^105^ Functionally, this descriptor mathematically integrates the geometric and electronic effects by coupling the coordination number (CN) of nearest-neighbor Pt atoms with an exponential strain term, while simultaneously accounting for ligand effects through the generalized coordination number (gCN) of surrounding Ni atoms. This data-driven formulation effectively disentangles the complex interplay between strain and ligand effects on realistic nanocatalyst surfaces. By applying this descriptor to the oxygen reduction reaction (ORR), a clear structure–activity relationship emerges, yielding a distinct volcano trend. Furthermore, comparative analysis against conventional geometric descriptors, such as the gCN, demonstrated the superiority of this ML-derived approach. On a data set of 39 DFT-calculated Pt atomic structures, the LED significantly outperformed gCN—both with and without strain corrections—in capturing binding energy variations, achieving a remarkably low root-mean-square error (RMSE) of 0.076 eV. These results underscore the critical role of ML in condensing complex, multidimensional structural features into unified descriptors that accurately predict catalytic performance.

Beyond selecting features from a predefined pool, AI can also function as a “mathematician″ to distill physical laws directly from data. Weng et al. applied symbolic regression (SR) to oxide perovskites to discover interpretable mathematical formulas that describe catalytic activity.^107^ As shown in Figure 8b, the algorithm generates a Pareto front that balances the complexity of the mathematical formula against its prediction error (MAE), allowing researchers to identify the optimal trade-off between accuracy and simplicity. From this frontier, a simple yet powerful descriptor, μ/*t* (where μ is the octahedral factor and *t* is the tolerance factor), was identified. Figure 8c demonstrates that this ratio exhibits a striking linear correlation with the experimentally measured OER activity (*V*_RHE_) across a wide range of perovskite compositions. This linear trend provides a transparent physical rule for rapid materials screening, circumventing the need for computationally expensive DFT calculations for every new candidate.

Complementing supervised methods, unsupervised learning offers a route to uncover hidden patterns in electronic structures without biased labels. Esterhuizen et al. utilized Principal Component Analysis (PCA) to extract electronic features directly from the density of states (DOS) of metal alloys.^108^ Unlike traditional descriptors that rely on specific moments like the d-band center, PCA captures the full shape and fine structure of the electronic states. Figure 8d illustrates the partial dependence plots derived from this analysis, revealing how the first principal component (PC1) quantitatively governs the adsorption energy trends for various species including C, O, N, and H. The plots show a monotonic relationship where increasing the PC1 score leads to stronger adsorption energies, demonstrating that unsupervised AI can extract physically meaningful electronic descriptors that unify the behavior of diverse adsorbates.

On the other hand, beyond automated feature discovery, explicitly extracting and analyzing specific physical features, particularly atomic ordering and disordering, provides a “white-box” pathway to elucidate catalytic mechanisms in solid-solution systems. Recent studies have highlighted that even with similar compositions, the spatial arrangement of atoms significantly dictates catalytic performance. For instance, Gong et al. investigated Pd_3_Bi alloys and revealed that the atomic arrangement serves as a critical geometric descriptor.^109^ Strikingly, experimental characterizations confirmed that these polymorphs possess nearly identical electronic structures; however, their catalytic behaviors diverge significantly. As shown in Figure 9a, the disordered solid solution facilitates oxidative C–H bond cleavage by offering optimized Pd ensemble sizes, thereby reducing the apparent activation energy (*E*_a_) compared to its ordered counterpart. The geometric origin of this enhancement is visually captured in Figure 9b, which contrasts the small, inactive Pd ensembles on the ordered surface (left) with the larger, catalytically active ensembles on the solid-solution surface (right). Moreover, their impact is not limited to simple geometric effects but extends to modulating catalytic performance through diverse physicochemical mechanisms such as electronic coupling and lattice strain. Yang et al. demonstrated that SRO induced by magnetic interactions in FeCoNiCuAl alloys can directly optimize the electronic structure of active sites.^110^ As illustrated in Figure 9c,d, the formation of specific SRO motifs modulates the d-band center to prevent the overbinding or weak-binding of intermediates like *H and *OH, effectively breaking the scaling relations and enhancing bifunctional water splitting activity via an electronic origin. In addition to electronic modulation, the degree of disorder can also influence the reaction pathway and structural robustness. Chen et al. utilized boron doping to disrupt the long-range crystallinity of RuO_2_, creating a highly disordered lattice.^111^ As depicted in Figure 9e, unlike the rigid crystalline RuO_2_ (top), where oxygen evolution is constrained by fixed lattice sites, the disordered A-RuO_2_ structure (bottom) offers structural flexibility. This flexibility modifies the coordination environment of Ru sites, thereby enabling a more favorable reaction pathway that facilitates oxygen evolution while suppressing the dissolution of active metal sites. Similarly, SRO can serve as a source of local lattice strain to enhance stability. Wang et al. incorporated short-range ordered Ta single atoms into a RuO_2_ matrix.^113^ Figure 9f explicitly illustrates the formation of specific Ru–O–Ta­(−O–Ta) motifs, which introduce significant tensile strain and create oxygen vacancies. This unique local structure accelerates the OER process, indicated by the “Fast″ cycle, compared to the “Slow″ cycle on conventional Ru–O–Ru sites. The Ta atoms act as electron donors to adjacent Ru sites via oxygen bridges, effectively suppressing the over-oxidation of Ru and stabilizing the active unit during acidic OER. Similarly, Zhao et al. recently revealed that introducing localized compressive strain by incorporating larger Ir atoms into a Ru-based HEA can efficiently modulate the electronic structure and strengthen interatomic interactions. This strain-induced effect shifts the OER pathway from the conventional adsorbate evolution mechanism (AEM) to the oxide pathway mechanism (OPM), thereby successfully breaking the activity–stability trade-off.^114^ These findings collectively underscore that “disorder″ in HEAs is not merely a random feature but a tunable parameter that can be engineered to optimize catalysis through geometric, electronic, and mechanical dimensions.

**Figure 9:**
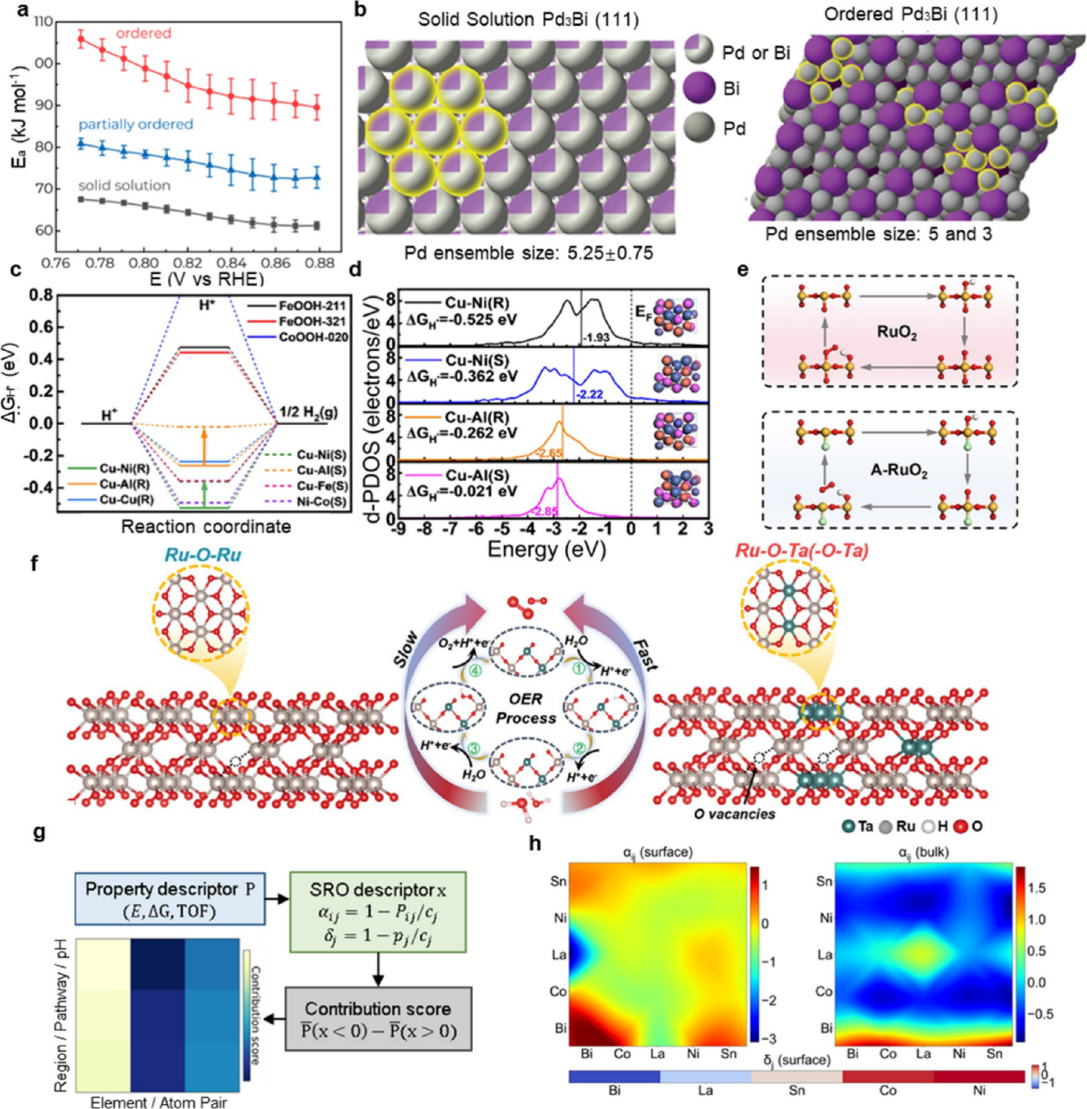
Mechanism elucidation of atomic ordering/disordering effects. (a and b) Geometric effect: Disordered solid-solution Pd_3_Bi exhibits lower activation energy (*E*_a_) than the ordered phase due to optimized surface Pd ensemble sizes. Reprinted with permission from ref 109. Copyright © 2025 American Chemical Society. (c and d) Electronic effect: SRO in FeCoNiCuAl HEAs modulates the d-band center to optimize hydrogen adsorption energetics (Δ*G*_H*_ ≈ 0). Reprinted with permission from ref 110. Copyright © 2024 Elsevier. (e and f) Structural flexibility and strain effects: Long-range disorder in B-doped RuO_2_ imparts structural flexibility to modify reaction pathways, while short-range ordered Ta atoms in RuO_2_ introduce lattice strain to stabilize active units via oxygen bridges. Reprinted with permission from ref 111. Copyright©2024 Wiley-VCH GmbH. (g and h) Quantitative decoupling of SRO contributions: Workflow for calculating SRO “contribution scores” by integrating Warren–Cowley (α_*ij*_) and LDD (δ_*j*_) parameters. Score heatmap quantifying the distinct impacts of surface and bulk SRO patterns on thermodynamic stability. Reproduced from ref 112. Available under a CC-BY 4.0 license. Copyright 2025 The Authors.

To achieve a more precise resolution of ordered or disordered effects within complex HEA spaces, Wu et al. recently proposed a quantitative framework to decouple the specific contributions of SRO to catalytic performance.^112^ By integrating the Warren–Cowley (W–C) parameters for pairwise correlations and Local Density Deviation (LDD) parameters for elemental distribution, they defined a statistical “contribution score” to quantify the distinct impact of local atomic environments as shown in Figure 9g. This methodology transforms the ambiguous “cocktail effect” into interpretable design rules. For example, as shown in Figure 9h, the framework successfully visualized the impact of SRO on thermodynamic stability for quinary La–Sn–Bi–Co-Ni nanoparticles. The score heatmap explicitly reveals that the stability is governed by specific SRO patterns, such as surface enrichment of Bi and the bulk enrichment of Co/Ni. This approach provides a systematic route to navigate the structure–performance landscape of complex catalysts, enabling the rational design of stable and active high-entropy materials. As research on HEA catalysts continues to develop, more universal methods or descriptors may emerge in the future, further advancing our understanding of these complex systems.

#### 5.2.2 Theoretical Evaluation of HER and ORR Performance

Experimental studies have established the bifunctional electrocatalytic activity of HEA nanoparticles; however, detailed mechanistic interpretation often depends on computational analyses that probe the electronic structure and reaction energetics. Accordingly, descriptor-based theoretical frameworks are commonly used to interpret and predict the HER and ORR performance of HEA catalysts.

For HER, descriptor-based theoretical analysis has traditionally depended on hydrogen adsorption free energy (Δ*G*_H*_) as the main measure of catalytic activity.^11,44^ According to the classical Sabatier principle, the best catalyst should interact with hydrogen in such a way that it is neither too strongly nor too weakly bound, which results in the well-known volcano correlation between Δ*G*_H*_ and HER activity.^115^ Nevertheless, HEAs are by design much more complex than single metal or binary catalysts in the nature of their catalytic activity. The difference in their highly heterogeneous atomic configurations leads to variations in local electronic structures, thus creating a broad distribution of adsorption strengths rather than a single characteristic Δ*G*_H*_. Due to this structural and electronic diversity, HER on HEAs cannot be sufficiently described by one single ΔG_H*_ value or the classical Sabatier volcano trend. Theoretically, different surface sites may serve different catalytic functions: strongly binding sites would be responsible for H* adsorption, while weakly binding ones would facilitate H_2_ release. In the event of fast surface diffusion, these different sites can operate in tandem, thus overriding the limitation of adsorption and desorption steps occurring on the same type of surface site.

By exploring the distinct surface complexity of HEAs, a recent work has put forward a mechanistic model called the “unusual Sabatier principle” that aims at describing HER intensity beyond the conventional single descriptor paradigm.^116^ As depicted in Figure 10a, the DFT calculations implemented on a systematic basis unveiled that imposing lattice strain on HEAs results in the gradual downshifts of the d band center, which points to a modifiable electronic structure. This electronic alteration is linked with hydrogen adsorption free energy (Δ*G*_H*_) changes that are usually referred to as the main descriptor for HER activity.

**Figure 10:**
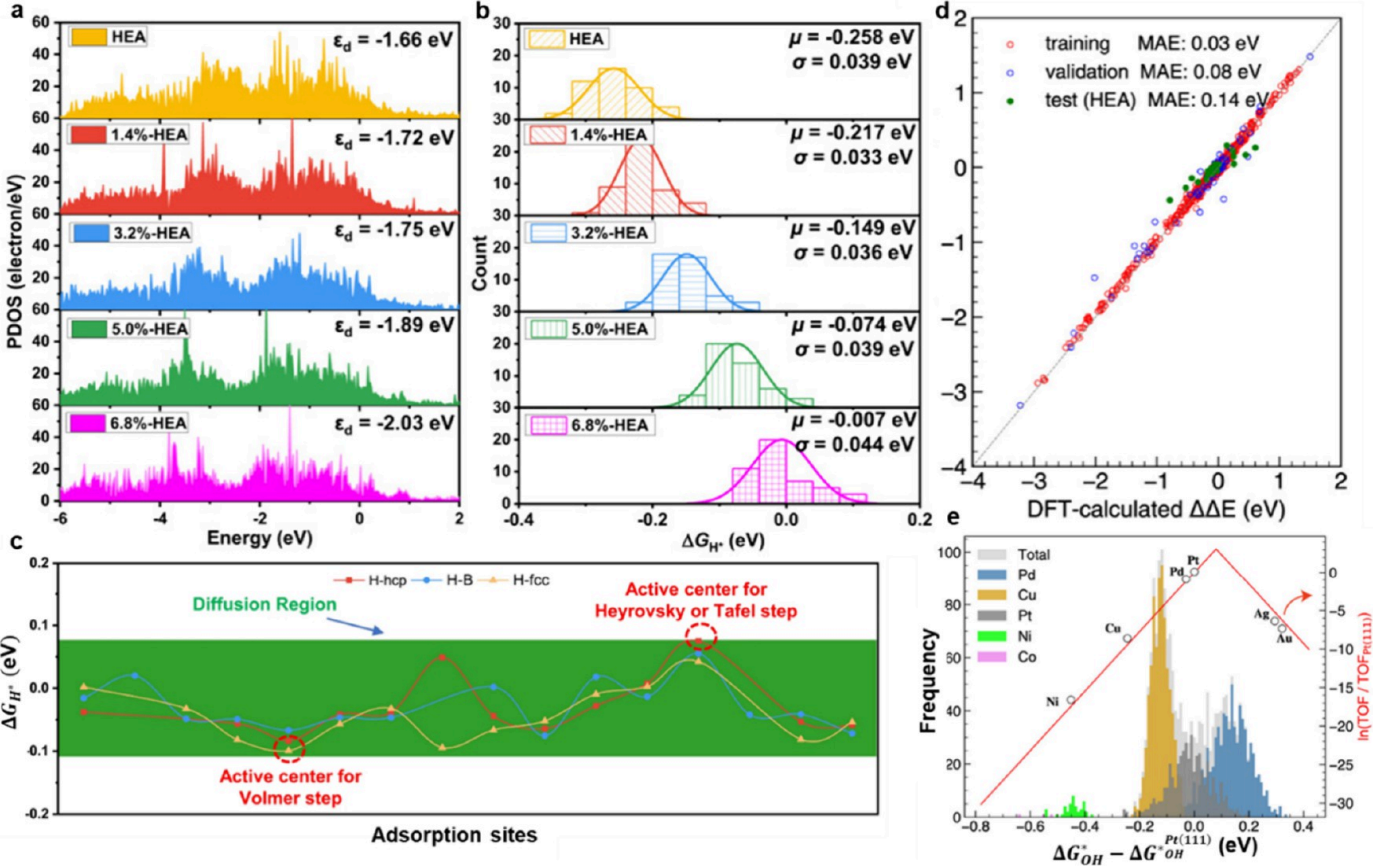
Electronic descriptors and theoretical models explaining HER and ORR activity in HEAs. (a) PDOS changes under lattice strain reveal gradual downshifts of the d band center (ε_d_), which directly regulate hydrogen adsorption energetics (Δ*G*_H*_). Reproduced from ref 116. Available under a CC-BY 4.0 license. Copyright 2024 Springer Nature. (b) Gaussian Δ*G*_H*_ distributions over HEA surfaces, described by the mean (μ) and standard deviation (σ). Catalysts with μ ≈ zero and larger σ show increased HER performance. Reproduced from ref 116. Available under a CC-BY 4.0 license. Copyright 2024 Springer Nature. (c) Δ*G*_H*_ spatial variation depicting the strongest binding sites for H* adsorption, the weakest binding sites for H_2_ release, and diffusion routes that allow multisite cooperative HER. Reproduced from ref 116. Available under a CC-BY 4.0 license. Copyright 2024 Springer Nature. (d) Machine learning results showing close physical correspondence with DFT calculated ΔΔ*E*_H*_, thus allowing a large-scale HER activity screening in diverse HEA configurations. Reproduced from ref 117. Available under a CC-BY 4.0 license. Copyright 2024 American Chemical Society. (e) Wide ranges of *OH adsorption free energies (ΔG_*OH_) for constituent metal environments, thus HEAs can accommodate multiple ORR active sites close to the volcano peak. Reproduced from ref 117. Available under a CC-BY 4.0 license. Copyright 2024 American Chemical Society.

However, instead of a single characteristic Δ*G*_H*_, different sites on HEA surfaces have a Gaussian distribution of Δ*G*_H*_ values, which is shown in Figure 10b. Two significant statistical parameters arise: the mean (μ), which represents the average adsorption strength, and the standard deviation (σ), which characterizes the range of adsorption site diversity. Catalysts with μ values close to 0 eV, corresponding to the best hydrogen binding, and larger σ values have better HER efficiency.^116^ This is consistent with the dual descriptor model, where both the energy of binding and the heterogeneity of adsorption sites are equally important.

In addition, the spatial Δ*G*_H*_ distribution over the HEA surface depicted in Figure 10c emphasizes the existence of different kinds of active centers. Strong binding sites (Δ*G*_H*_ < 0 eV) are sites for H adsorption, whereas weak binding sites (Δ*G*_H*_ > 0 eV) are places where H_2_ is released through Heyrovsky or Tafel steps. The sites are connected via a low-barrier diffusion region (∼0.124 eV), which enables hydrogen atoms to move from one site to another. This cooperative multisite mechanism is thus a very efficient way of separating adsorption and desorption steps, thereby accelerating HER kinetics. Combined with the classical Sabatier volcano, this model extends the latter by introducing (μ, σ) as a dual descriptor instead of a single descriptor.^116^ It is better at representing HER on HEAs and, therefore, provides invaluable insight into the design of the next generation of HER catalysts that would exploit surface complexity and spatially resolved activity.

In comparison to HER, ORR shows a significantly more complicated theoretical scenario due to its multielectron, multistep reaction pathway, which involves several oxygenated intermediates such as *O_2_, *OOH, *O, *OH. Even though DFT offers a very solid base for calculating the binding energetics of these species, its direct use with HEAs is very much constrained.^11,32,118^ The reason is that HEA surfaces have millions of different local atomic environments, and the adsorption energy of oxygen intermediates is highly dependent on the exact coordination of each site. Additionally, for HEA compositions containing magnetic elements (e.g., Fe, Co, Ni, Mn), spin polarization may significantly influence the local electronic structure and adsorption energetics. Neglecting these magnetic moments in first-principles calculations may lead to inaccurate predictions of intermediate binding strengths, particularly for oxygenated species active in the ORR.^110^ Therefore, a brute force DFT sampling is computationally very expensive if one wants to map the entire reactivity landscape of HEAs.^119^ For example, sampling on the order of 10^4^ distinct surface local environments for adsorption studies would typically require ∼10^5^–10^6^ CPU hours with conventional DFT, making brute-force enumeration impractical.

To mitigate this difficulty, a couple of recent papers have put forward DFT-integrated ML frameworks that basically open the way for a quick evaluation of ORR activity on HEAs. A typical tactic is the DFT + ML high-throughput screening method, where *OH adsorption energies calculated by DFT on a very few sites are used to create predictive models that can estimate ΔG_*OH_ for the vast number of possible adsorption environments.^120^ Another convincing method is the one that uses deep learning interatomic potentials together with Monte Carlo (MC) simulations for the production of compositional HEA surface configurations that are as close to reality as possible. After that, these structures are evaluated by physics-guided ML models like TinNet that can locate adsorption energies with almost DFT accuracy.^117^ TinNet, as depicted in Figure 10d, is in very close agreement with DFT (MAE ≈ 0.14 eV for HEA test sets) and thus allows, in principle, unlimited sampling of complex HEA surfaces at a huge scale.^117^ Among the computational approaches discussed, the *OH adsorption free energy (ΔG_*OH_) has emerged as the most robust and transferable single descriptor for evaluating ORR activity.^121^ Owing to the well-established linear scaling relations among oxygenated intermediates (*O_2_, *OOH, *O, and *OH), ΔG_*OH_ alone can effectively reconstruct the ORR volcano relationship and capture the classical Sabatier-type balance between intermediate binding strength and catalytic turnover.^117^ As illustrated in Figure 10e, HEAs intrinsically generate a broad distribution of ΔG_*OH_ values as a direct consequence of their highly diverse local atomic environments. Rather than exhibiting a single characteristic adsorption strength, a single HEA surface hosts a wide spectrum of adsorption sites spanning weak-, intermediate-, and strong-binding regimes. Importantly, this broad distribution enables multiple surface motifs within the same catalyst to reside at or near the peak of the ORR volcano, providing a molecular-level explanation for the superior ORR performance frequently observed in HEA systems.^117^ And Figure 10e also reveals that adsorption sites associated with elements such as Pt and Pd predominantly populate the optimal binding region close to the volcano maximum, whereas Cu- and Ni-centered environments tend to shift ΔG_*OH_ toward either weaker or stronger binding regimes.^117^ This element-resolved distribution highlights how HEAs combine chemically distinct local environments within a single solid-solution framework, thereby achieving simultaneous access to multiple ORR-active configurations that are difficult to realize on compositionally uniform surfaces.^102,117^ Collectively, these results demonstrate that the ORR activity of HEAs is best understood from a statistical and site-distribution perspective, where catalytic performance arises from the coexistence of numerous near-optimal adsorption sites rather than from a single “ideal” active center. In this context, the integration of DFT calculations with machine-learning–accelerated surface sampling provides a practical and scalable route to map adsorption-energy landscapes and to rationally screen high-entropy electrocatalysts for oxygen reduction.

From a catalyst design point of view, these results imply that the next generation of HEA electrocatalysts should not just focus on trying to achieve a single ideal adsorption energy. On the contrary, rational design approaches should be based on the modification of the statistical features of the adsorption and energy distributions.

In the case of HER, it is about setting the distribution center (μ) of G_*H_ to the thermoneutral binding condition and at the same time increasing the distribution width (σ) to raise the fraction of hydrogen, binding sites with near, optimal energies, thus allowing cooperative adsorption and desorption over several surface motifs. Similarly, for ORR, the oxygenated, intermediate adsorption energies (especially G_*OH_) distribution needs to be modified so that a large portion of the surface sites would fall close to the ORR volcano maximum instead of optimizing a single site only.

In the case of bifunctional HEA catalysts, these distribution-based design rules point out the necessity of simultaneously satisfying different but complementary adsorption requirements of HER and ORR related intermediates. By adjusting composition, strain engineering, and intentional creation of local atomic environments, one can fine-tune both the location and size of adsorption, energy distributions, thus opening up the maximum number of catalytically active sites for each reaction and achieving real bifunctionality in a single material.

#### 5.2.3 Performance Evaluation for Bifunctional Catalysts

Designing bifunctional catalysts for HER and ORR introduces additional complexities, as these catalysts must balance the performance requirements for two distinct reactions. The optimization process is particularly challenging in two aspects. The first difficulty lies in designing standards. Unlike single-function catalysts, bifunctional catalysts currently lack a standardized metric for evaluating their overall performance. Although catalysts that exhibit excellent performance in both HER and ORR have been successfully synthesized experimentally, from the computational design perspective, the ability to rapidly model different structures is undoubtedly an advantage.^26^ However, to achieve more efficient screening and performance optimization, it is essential to establish more rational and targeted design standards. Developing a unified descriptor or performance index that accounts for both reactions remains a significant challenge. A second challenge arises from the intrinsic complexity of bifunctional catalyst design. Achieving stability, activity, and selectivity for a single electrochemical reaction is already nontrivial; satisfying these requirements simultaneously for both HER and ORR further increases the design difficulty. The two reactions impose distinct kinetic and thermodynamic constraints, and optimization toward one pathway can readily compromise the other. For instance, surfaces optimized for near-thermoneutral hydrogen adsorption (Δ*G*_H*_ ≈ 0) to promote HER may bind oxygenated intermediates too strongly, thereby suppressing ORR activity. While the diverse local atomic environments in HEAs enable bifunctionality, they also complicate rational design, as motifs favorable for hydrogen adsorption can hinder oxygen reduction.

Recent advances in computational catalyst design suggest that multiobjective optimization strategies provide a viable route to addressing these challenges.^79^ Rather than identifying a single optimal descriptor, these approaches define a set of trade-off solutions, commonly represented by a Pareto front, where improvement in one objective cannot be achieved without degrading another.^115^ Recent studies have addressed this. For instance, Xu et al. developed a Pareto-based optimization framework to explore the vast compositional space of HEAs, enabling simultaneous optimization of catalytic activity and structural stability. By training machine-learning models to predict key descriptors such as Δ*G*_OH_ and surface formation energies across millions of candidate configurations, the authors identified alloy compositions that achieve balanced performance.^122^ This framework enables concurrent tuning of hydrogen adsorption free energy (Δ*G*_H*_) governing HER and hydroxyl adsorption free energy (Δ*G*_OH*_) governing ORR, providing a systematic pathway toward bifunctional optimization.^5,23^ Despite these advances, challenges remain, including accurate modeling of local atomic environments, treatment of intermediate cross-reactivity, and the limited availability of reliable data sets encompassing multiple reaction mechanisms in complex HEA systems.

It is essential to distinguish between descriptors that primarily govern intrinsic catalytic activity and those that more accurately reflect long-term structural stability. Activity-related descriptors, such as adsorption energetics and electronic structure parameters, are typically used to rationalize catalytic performance under simplified and idealized atomic environments. In contrast, stability-related descriptors are more closely associated with phenomena occurring under realistic operating conditions, including surface reconstruction, elemental segregation, and thermodynamic driving forces. These aspects are discussed in greater detail in the following section.

### 5.3 Realistic Electrochemical Environment Effects: Solvent, Electric Field, pH, and Catalyst Stability

Density functional theory (DFT) calculations have played a central role in elucidating fundamental electrocatalytic mechanisms. However, most conventional DFT studies are performed under idealized vacuum or gas-phase conditions, which neglect the complex and dynamic nature of the solid–liquid interface where electrochemical reactions occur. This interface comprises solvent molecules, the electric double layer (EDL), interfacial electric fields, and pH-dependent ionic species, all of which can substantially influence reaction energetics and pathways.^123^ These limitations are particularly pronounced for bifunctional electrocatalysts such as HEAs, whose chemically heterogeneous surfaces host multiple competing active sites. In such systems, even modest perturbations arising from local electric fields, proton availability, or solvent stabilization can lead to pronounced changes in activity and selectivity, rendering static, vacuum-based DFT descriptions insufficient and often inconsistent with experimental observations.^124,125^ These limitations are particularly pronounced for bifunctional electrocatalysts such as HEAs, whose chemically heterogeneous surfaces host multiple competing active sites. In such systems, even modest perturbations arising from local electric fields, proton availability, or solvent stabilization can lead to pronounced changes in activity and selectivity, rendering static, vacuum-based DFT descriptions insufficient and often inconsistent with experimental observations.

A primary consequence of the electrochemical environment is the applied electrode potential, which generates a strong interfacial electric field across the Helmholtz layer.^126^ This field directly modulates the energetics, dipole moments, and orientations of adsorbed intermediates. Importantly, electric field effects are intrinsically coupled to pH, as reaction mechanisms depend on the interplay between interfacial fields and proton availability. Acidic conditions typically favor concerted proton–electron transfer (CPET), whereas alkaline environments often promote stepwise electron-transfer or proton-transfer pathways due to kinetic decoupling.^127,128^ To capture these effects and rationalize experimentally observed trends such as pH-dependent Tafel slopes, constant-potential approaches where the electrode is treated as an electron reservoir, are generally more appropriate than fixed-charge models.^129,130^ These methods explicitly account for field–adsorbate interactions, which are especially critical for ORR selectivity, where the stability of polar intermediates such as *OOH is sensitive to field orientation and magnitude.

Beyond activity, catalyst stability under operando conditions represents an equally important yet frequently underexplored aspect of computational screening.^131^ Although Pourbaix analysis provides a useful thermodynamic framework for identifying stability windows and dissolution tendencies, thermodynamics alone is insufficient.^132^ Kinetic factors, including dissolution barriers, surface segregation, and reconstruction pathways, must also be considered. In HEAs, configurational entropy stabilizes the bulk phase, but surfaces remain vulnerable to selective leaching, oxidation-driven reconstruction, or dynamic segregation. As a result, adsorption energetics can evolve with time, implying that descriptors derived from pristine surfaces may not reliably predict long-term catalytic performance. This dynamic behavior highlights the need for stability-aware modeling strategies in HEA catalyst design. Beyond activity, catalyst stability under operando conditions represents an equally important yet frequently underexplored aspect of computational screening. Although Pourbaix analysis provides a useful thermodynamic framework for identifying stability windows and dissolution tendencies, thermodynamics alone is insufficient. Kinetic factors, including dissolution barriers, surface segregation, and reconstruction pathways, must also be considered. In HEAs, configurational entropy stabilizes the bulk phase, but surfaces remain vulnerable to selective leaching, oxidation-driven reconstruction, or dynamic segregation. As a result, adsorption energetics can evolve with time, implying that descriptors derived from pristine surfaces may not reliably predict long-term catalytic performance. This dynamic behavior highlights the need for stability-aware modeling strategies in HEA catalyst design.

Finally, the solvent itself actively participates in electrocatalysis rather than serving as a passive dielectric medium.^133,134^ Solvent molecules stabilize charged intermediates, mediate proton transfer through hydrogen-bonding networks, and can significantly lower reaction barriers.^135^ For instance, explicit water stabilization of *OH on HEA surfaces can alter desorption kinetics and shift reaction pathways.^136^ To capture these effects, computational studies increasingly employ implicit solvation models to describe average electrostatic screening, as well as explicit approaches such as ab initio molecular dynamics (AIMD) to resolve interfacial fluctuations and solvent–adsorbate interactions directly.^137^ Incorporating these realistic environmental factors is therefore essential for achieving reliable predictions of both activity and durability, ultimately enabling the rational design of HEA-based bifunctional electrocatalysts. Finally, the solvent itself actively participates in electrocatalysis rather than serving as a passive dielectric medium. Solvent molecules stabilize charged intermediates, mediate proton transfer through hydrogen-bonding networks, and can significantly lower reaction barriers. For instance, explicit water stabilization of *OH on HEA surfaces can alter desorption kinetics and shift reaction pathways. To capture these effects, computational studies increasingly employ implicit solvation models to describe average electrostatic screening, as well as explicit approaches such as AIMD to resolve interfacial fluctuations and solvent–adsorbate interactions directly. Incorporating these realistic environmental factors is therefore essential for achieving reliable predictions of both activity and durability, ultimately enabling the rational design of HEA-based bifunctional electrocatalysts.

However, the application of these realistic models is constrained by significant computational costs. Constant-potential methods, for instance, require iterative optimization of the surface charge to maintain the target Fermi level, which can increase the simulation time by an order of magnitude compared to standard constant-charge calculations.^123,128^ Similarly, the choice of solvent model presents a critical trade-off: while implicit models provide a computationally efficient description of dielectric screening, they fail to capture the specific hydrogen-bonding networks that are essential for stabilizing intermediates like *OH.^132,137^ Conversely, explicit solvent models combined with ab initio molecular dynamics (AIMD) offer high accuracy but are often too computationally expensive for the high-throughput screening required to explore the vast HEA configurational space. Therefore, a hierarchical sampling strategy, utilizing static DFT with implicit solvation for broad compositional screening, followed by targeted AIMD simulations for precise mechanistic verification, is typically the most practical approach.^123^

Beyond computational expense, these models introduce physical approximations that limit quantitative accuracy. Constant-potential and electric-field approaches typically treat the EDL within a mean-field continuum framework, which is insufficient for HEA surfaces where strong electronic heterogeneity produces highly localized, nonuniform electric fields.^123–125^ In addition, pH-dependent models such as the Computational Hydrogen Electrode (CHE) assume thermodynamic equilibrium for proton–electron transfer, reducing reliability under kinetic regimes, particularly in alkaline media or at high overpotentials, where decoupled transfer steps and site-specific solvation environments create barriers not captured by bulk thermodynamic corrections.^127,128^

## 6 Challenge and Opportunity

HEAs are fast becoming one of the most fascinating, though very challenging, bifunctional electrocatalysis platforms. On the one hand, their compositional variety and inherent structural disorder provide the possibility of meeting the catalytic needs of the HER and the ORR simultaneously. On the other hand, they pose the problem of how to unify these requirements. We put forward the idea of first addressing three research directions that can not only convert the intricacy of HEAs into a strong design leverage but also clarify the confusion.

First of all, developing artificial intelligence driven strategies for the design of the HEA catalyst is the primary technological advancement that should be accompanied by the establishment of proper data infrastructures. These data infrastructures should be specially constructed for the disordered multicomponent systems as well as bifunctional performance targets. The extremely large compositional design space, short-range order, the heterogeneity of active sites, and dynamic surface reconstruction under the working conditions make conventional brute force simulations or purely empirical screening infeasible. Hence, data-centric methodologies become the only efficient and accurate way of dealing with such complexity. While crystalline catalyst databases become more and more mature, HEA data sets are still very limited and often inconsistent. In general, they do not comprise paired HER and ORR activity metrics; standardized testing such as electrolyte type or pH; durability information; or details about catalyst supports and interfaces. A HEA database devoted to this purpose should incorporate structural descriptors at different scales that include elemental composition, short-range ordering patterns, coordination number distributions, strain statistics, and key electronic characteristics. These descriptors should be connected with catalytic metrics such as hydrogen adsorption free energy ΔG_H*_, oxygenated intermediate binding energies including *OOH and *O, half wave potentials, Tafel slopes, mass activities, and stability measurements. Hybrid workflows that include rapid surrogate models and uncertainty-aware active learning, as well as targeted high-accuracy DFT simulations and operando experiments, will be necessary to strike a balance between computational cost and predictive fidelity. The establishment of shared data standards, benchmarking protocols, and traceable metadata practices is not only essential for data reliability but also serves as a basis for the mechanistic and closed-loop strategies discussed below.

Second, it is a prerequisite to comprehend the cooperative and synergistic interactions in HEAs at a deeper mechanistic level in order to have a catalyst design that is interpretable and directional. Nonlinear interactions among the constituent elements are mostly responsible for the catalytic behavior of HEAs. These interactions manifest as the coupling of electronic and geometric, local lattice strain, broadened adsorption energy distributions, and interfacial charge redistribution. It is impossible to derive these effects reliably from the trends established for single-component catalysts. In the case of bifunctional operation, a design problem arises: on the one hand, HER performance necessitates ΔG_H*_ optimization, on the other hand, ORR performance depends on the energetics of intermediates such as *OOH and *O. In fact, these requirements have to be reconciled over a broad distribution of active sites whose identities and electronic states may change under the applied potential. Interpretable data driven models such as symbolic regression, SISSO type descriptor identification, and graph based learning frameworks with attribution capabilities ought to form connections between structurally measurable motifs in the experiment such as short-range ordering, local coordination geometries, and gradients in electronegativity or d band characteristics, and mechanistic indicators like rate limiting barriers, selectivity trends, and the relationships between activity and stability. These models ought to be strongly coupled with operando characterization tools such as X-ray absorption spectroscopy, Raman spectroscopy, electrochemical transmission, or scanning tunneling microscopy, and online mass spectrometry. The coupling makes it possible to determine the real working active sites, the main reconstruction pathways, and the sources of deactivation. Hence, empirical correlations can be turned into testable causal hypotheses that explain how HEAs achieve bifunctional performance.

Third, after enhancing the progress not only in the data infrastructure but also in the mechanistic understanding, the industry should embrace a closed-loop design system that unites theory, synthesis, characterization, and electrochemical evaluation without any interruption. In this system, predictive models with quantified uncertainty identify candidate HEA compositions, synthesis pathways, and targeted short-range ordering motifs. Automated or high-throughput synthesis platforms and standardized HER and ORR measurement protocols can then create consistent experimental data. All this data, including the unsuccessful attempts, is the learning framework’s feedback to refine descriptors, improve model accuracy, and update prior assumptions. Multiobjective optimization may be used to balance catalytic activity, selectivity, long-term durability, and practical constraints involving elemental cost, corrosion resistance, and resource availability. For instance, recent work on scalable MOF-based electrodes has demonstrated the critical value of integrating techno-economic analysis (TEA) and life cycle assessment (LCA) early in the design phase to quantify hydrogen production costs and environmental carbon footprints, setting a benchmark for practical catalyst development.^138^ Synthetic feasibility-related constraints are considered through physics-based surrogate models. The iteration cycle can be shortened dramatically with the increased use of autonomous experimentation and standardized data practices, thus enabling the rapid and interpretable development of HEA catalysts. Now is the time to realize this AI-guided, mechanism-informed, closed-loop design paradigm. The way it is implemented can turn HEAs into practical and scalable bifunctional catalysts that can meet the strict performance requirements of both HER and ORR, instead of being highly complex research curiosities.
